# Extended Analysis of HIV Infection in Cisgender Men and Transgender Women Who Have Sex with Men Receiving Injectable Cabotegravir for HIV Prevention: HPTN 083

**DOI:** 10.1128/aac.00053-23

**Published:** 2023-03-30

**Authors:** Mark A. Marzinke, Jessica M. Fogel, Zhe Wang, Estelle Piwowar-Manning, Ryan Kofron, Amber Moser, Pradip Bhandari, Ryann Gollings, Lane R. Bushman, Lei Weng, Elias K. Halvas, John Mellors, Peter L. Anderson, Deborah Persaud, Craig W. Hendrix, Marybeth McCauley, Alex R. Rinehart, Marty St Clair, Susan L. Ford, James F. Rooney, Adeola Adeyeye, Suwat Chariyalertsak, Kenneth Mayer, Roberto C. Arduino, Myron S. Cohen, Beatriz Grinsztejn, Brett Hanscom, Raphael J. Landovitz, Susan H. Eshleman

**Affiliations:** a Department of Pathology, Johns Hopkins University School of Medicine, Baltimore, Maryland, USA; b Department of Medicine, Johns Hopkins University School of Medicine, Baltimore, Maryland, USA; c Fred Hutchinson Cancer Research Center, Seattle, Washington, USA; d Department of Medicine, University of California, Los Angeles, Los Angeles, California, USA; e Department of Pharmaceutical Sciences, University of Colorado, Aurora, Colorado, USA; f Department of Medicine, University of Pittsburgh, Pittsburgh, Pennsylvania, USA; g Department of Pediatrics, Johns Hopkins University School of Medicine, Baltimore, Maryland, USA; h Department of Clinical Pharmacology, Johns Hopkins University School of Medicine, Baltimore, Maryland, USA; i FHI 360, Durham, North Carolina, USA; j ViiV Healthcare, Research Triangle Park, North Carolina, USA; k GlaxoSmithKline, Research Triangle Park, North Carolina, USA; l Gilead Sciences, Foster City, California, USA; m Prevention Science Program, Division of AIDS, National Institute of Allergy and Infectious Diseases, National Institutes of Health, Rockville, Maryland, USA; n Research Institute for Health Sciences, Chiang Mai University, Chiang Mai, Thailand; o The Fenway Institute, Fenway Health, Boston, Massachusetts, USA; p Department of Medicine, Harvard Medical School, Boston, Massachusetts, USA; q Department of Internal Medicine, McGovern Medical School, University of Texas Health Science Center at Houston, Houston, Texas, USA; r Department of Medicine, University of North Carolina at Chapel Hill, Chapel Hill, North Carolina, USA; s Instituto de Pesquisa Clinica Evandro Chagas-Fiocruz, Rio de Janeiro, Brazil; t Center for Clinical AIDS Research and Education, University of California, Los Angeles, Los Angeles, California, USA

**Keywords:** HIV, preexposure prophylaxis, prevention, cabotegravir, injectable, TDF-FTC, long-acting, men who have sex with men, HPTN 083

## Abstract

HPTN 083 demonstrated that injectable cabotegravir (CAB) was superior to oral tenofovir disoproxil fumarate-emtricitabine (TDF-FTC) for HIV prevention in cisgender men and transgender women who have sex with men. We previously analyzed 58 infections in the blinded phase of HPTN 083 (16 in the CAB arm and 42 in the TDF-FTC arm). This report describes 52 additional infections that occurred up to 1 year after study unblinding (18 in the CAB arm and 34 in the TDF-FTC arm). Retrospective testing included HIV testing, viral load testing, quantification of study drug concentrations, and drug resistance testing. The new CAB arm infections included 7 with CAB administration within 6 months of the first HIV-positive visit (2 with on-time injections, 3 with ≥1 delayed injection, and 2 who restarted CAB) and 11 with no recent CAB administration. Three cases had integrase strand transfer inhibitor (INSTI) resistance (2 with on-time injections and 1 who restarted CAB). Among 34 CAB infections analyzed to date, diagnosis delays and INSTI resistance were significantly more common in infections with CAB administration within 6 months of the first HIV-positive visit. This report further characterizes HIV infections in persons receiving CAB preexposure prophylaxis and helps define the impact of CAB on the detection of infection and the emergence of INSTI resistance.

## INTRODUCTION

Cabotegravir (CAB) is a potent integrase strand transfer inhibitor (INSTI). A long-acting injectable form of CAB (CAB-LA) was recently approved by the U.S. Food and Drug Administration (FDA) to reduce the risk of sexual HIV transmission based on the results of two clinical trials that demonstrated that CAB-LA was superior to oral tenofovir disoproxil fumarate-emtricitabine (TDF-FTC) for HIV preexposure prophylaxis (PrEP): HPTN 083 and HPTN 084 (1, 2). HPTN 083 enrolled cisgender men and transgender women (TGW) who have sex with men at 43 study sites in the United States, Latin America, Africa, and Asia (1). After the trial was unblinded, participants received the randomly assigned study regimen without placebo until the protocol was amended. Study sites transitioned to an open-label extension (OLE) starting in April 2021; in the OLE, participants could continue their original randomized PrEP regimen or switch to the other regimen.

We previously characterized HIV infections in the blind phase of HPTN 083 (3, 4). We found that the detection of infection was often delayed at study sites in persons with recent CAB administration (3). We used a low-viral-load INSTI genotyping assay to evaluate the timing of the emergence of INSTI resistance (4). We found that an HIV RNA assay with a lower limit of detection (LLOD) of 30 copies/mL detected most infections before INSTI resistance emerged (4).

This report extends our previous work by characterizing HIV infections that occurred through the end of the first unblinded year of HPTN 083. This builds on our previous studies characterizing CAB arm infections and provides additional information on the detection of HIV infection and the emergence of INSTI resistance in this setting. It also provides additional information on the pharmacokinetics of CAB-LA PrEP.

(Some of the data in this report were presented at the Conference on Retroviruses and Opportunistic Infections [February 2022] and the HIV Glasgow Meeting [October 2022]).

## RESULTS

### Case classification.

Fifty-eight HIV infections were previously identified in the blinded phase of HPTN 083 (16 in the CAB arm and 42 in the TDF-FTC arm). Fifty-two additional cases are described in this report (18 in the CAB arm and 34 in the TDF-FTC arm); 3 of these infections occurred during the blinded phase of the trial (1 in the CAB arm and 2 in the TDF-FTC arm). CAB arm infections were classified into 6 groups (A, B, C, D, DX, and BR) based on the relationship between CAB administration and the first HIV-positive visit (Table 1). TDF-FTC arm infections were assigned to group E; these cases are described in File S2 in the supplemental material. The 18 new CAB arm infections include 5 cases with recent CAB administration and 2 cases where CAB was restarted after infection. These 7 cases are described below; HIV test results from the study sites and the HIV Prevention Trials Network (HPTN) Laboratory Center are provided in File S3. The 11 cases with no recent CAB administration are described in File S4.

**TABLE 1:** Classification of HIV infections in the CAB arm of HPTN 083^*a*^

Case type	Case description	Infection description	Classification		No. of cases
			Previous report	Current report	
A	Baseline infection	HIV positive at study enrollment	A1–A4		4
B	No recent CAB administration	No CAB administration in the 6 mo before the 1st HIV-positive visit	B1–B5	B6–B16	16
C	Oral-phase infection	Infected during the 5-wk oral CAB lead-in phase	C1–C3		3
D	Infected despite on-time injections	Infected <6 mo after the last injection with on-time injections	D1–D4^*b*^	D5–D6	6
DX	Delayed injection	Infected <6 mo after the last injection with at least 1 delayed injection (>70 days after the last injection)		DX1–DX3	3
BR	CAB restarted after infection	No CAB administration in the 6 mo before the 1st HIV-positive visit; CAB restarted at or after the 1st HIV-positive visit		BR1–BR2	2

Total no. of cases			16	18	34

a The approach used to classify infections in the cabotegravir (CAB) arm of HPTN 083 is shown. Sixteen cases were described in a previous report (3); 18 additional cases are included in this report. HIV testing performed at study sites and the HPTN Laboratory Center was used to determine HIV infection status and identify the first HIV-positive visit. Injections were classified as delayed if they were administered >2 weeks after the planned injection date (>44 days after the 1st injection or >70 days after subsequent injections).

b One case that was classified as a D case in the previous report had a single late injection (D1) (75 days after the previous injection); that case would have been classified as a DX case according to the updated classification system used in this report.

### CAB arm infections.

**FIG 1:**
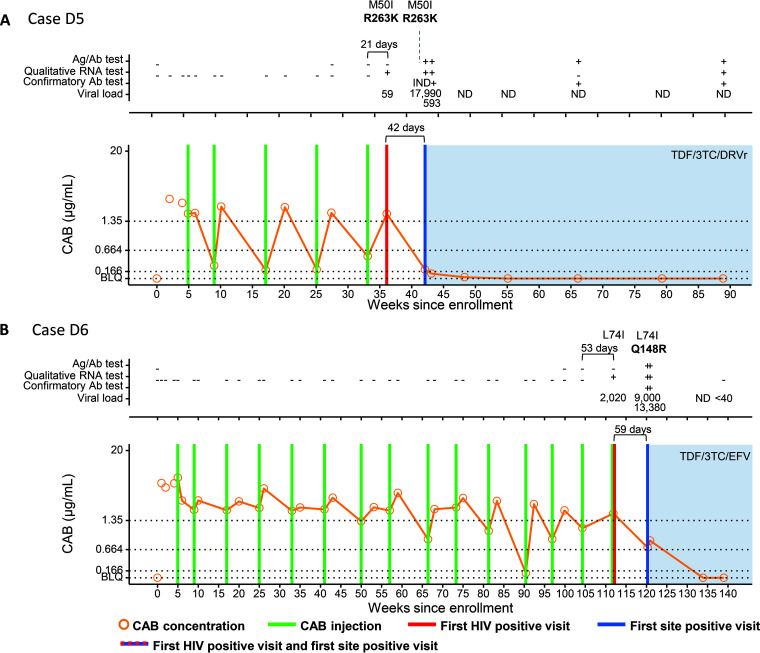
Summary of key events and laboratory results for participants in the cabotegravir (CAB) study arm of HPTN 083: cases D5 (A) and D6 (B). Results from testing performed at study sites are shown in File S2 in the supplemental material. Test results and key study events are shown as a function of the number of weeks from study enrollment based on calendar dates. Results obtained from testing performed at the HPTN Laboratory Center are shown above each graph. Positive and reactive results are indicated with a plus sign; negative and nonreactive results are indicated with a minus sign. IND indicates an indeterminate test result. VL values are shown (number of HIV RNA copies per milliliter); results noted as <40 indicate that HIV RNA was detected at a level below 40 copies/mL. Results from HIV drug resistance testing are shown. All drug resistance mutations are shown for INSTIs; major INSTI mutations are shown in boldface type. The brackets at the top of the graphs show the number of days between the last injection and the first HIV-positive visit. The lower brackets show the number of days between the first site-positive visit and the first HIV-positive visit for cases where there was a delay in site detection. The graphs show plasma CAB concentrations and key events. The key at the bottom describes the symbols used in the graphs. Horizontal lines indicate the following concentration cutoffs: 1.33 μg/mL for 8× PA-IC_90_ (*in vitro* protein-adjusted 90% CAB inhibitory concentration), 0.664 μg/mL for 4× PA-IC_90_, and 0.166 μg/mL for 1× PA-IC_90_. BLQ indicates that the CAB concentration was below the limit of quantification (<0.025 μg/mL). Shaded areas indicate that the participant was on antiretroviral therapy. 3TC, lamivudine; Ag/Ab, antigen/antibody; DRVr, ritonavir-boosted darunavir; EFV, efavirenz; ND, not detected; TDF, tenofovir disoproxil fumarate.

**(i) Infections that occurred despite on-time CAB injections (D cases).** Participant D5 acquired HIV infection during the blinded phase of the study (Fig. 1). This participant received five on-time CAB injections before the first HIV-positive visit, which was 21 days after the last CAB injection. At the first HIV-positive visit, the viral load (VL) was 59 copies/mL. The site detected the infection 42 days later. The CAB concentration at the first HIV-positive visit was 1.906 μg/mL. The major INSTI resistance-associated mutation (RAM) R263K was detected at the first HIV-positive visit, 21 days after the last injection. The CAB concentration-time profile in this case indicates consistent on-time dosing with rapidly declining CAB concentrations after typical peak concentrations. Low CAB trough concentrations, all <4× the *in vitro* protein-adjusted 90% CAB inhibitory concentration (PA-IC_90_) (range, 0.198 to 0.523 μg/mL), were observed. This is likely attributed to the high CAB-LA absorption rate constant, *k_a_*, which is estimated to range from 1.5 × 10^−3^ to 3.9 × 10^−3^ h^−1^ in this participant and is increased from the population-based mean *k_a_* of 7.3 × 10^−4^ h^−1^ reported in a previous multistudy analysis (5). The participant’s body mass index (BMI) of 24 kg/m^2^, which is just below the population mean across numerous CAB studies, has little influence on *k_a_* in this participant (5–7).

Participant D6 had 14 on-time CAB injections before the first HIV-positive visit (Fig. 1). An additional injection was given at the first HIV-positive visit. The major INSTI RAM Q148R was detected 59 days after the first HIV-positive visit, which was the first visit where the site detected the infection. The CAB concentration at the first HIV-positive visit was 1.824 μg/mL; CAB concentrations were ≥4× PA-IC_90_ and ≥8× PA-IC_90_ at 97% and 83% of the previous study visits, respectively. However, five of the six trough CAB concentrations immediately preceding the first HIV-positive visit were <8× PA-IC_90_, including one CAB concentration that was <2× PA-IC_90_. The VL was 2,020 copies/mL at the first HIV-positive visit.

**FIG 2:**
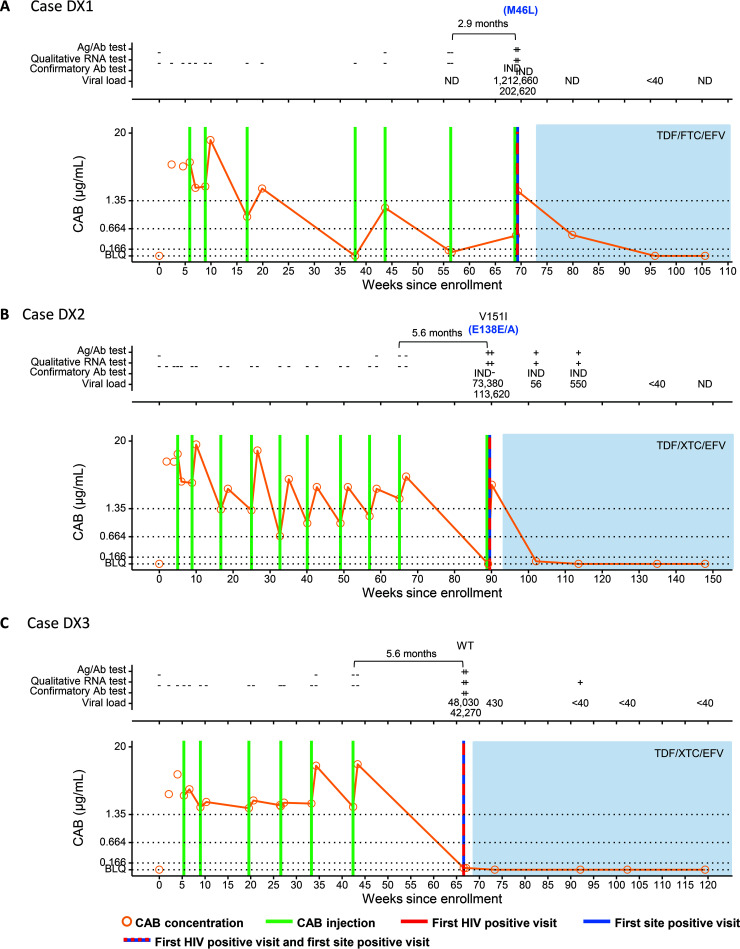
Summary of key events and laboratory results for participants in the cabotegravir (CAB) study arm of HPTN 083: cases DX1 (A), DX2 (B), and DX3 (C). Results from testing performed at study sites are shown in File S2 in the supplemental material. Test results and key study events are shown as a function of the number of weeks from study enrollment based on calendar dates. Results obtained from testing performed at the HPTN Laboratory Center are shown above each graph. Positive and reactive results are indicated with a plus sign; negative and nonreactive results are indicated with a minus sign. IND indicates an indeterminate test result. VL values are shown (number of HIV RNA copies per milliliter); results noted as <40 indicate that HIV RNA was detected at a level below 40 copies/mL. Results from HIV drug resistance testing are shown. All drug resistance mutations are shown for INSTIs. Major drug resistance mutations for other drug classes are shown in blue text in parentheses. The brackets at the top of the graphs show the number of days between the last injection and the first HIV-positive visit. The graphs show plasma CAB concentrations and key events. The key at the bottom describes the symbols used in the graphs. Horizontal lines indicate the following concentration cutoffs: 1.33 μg/mL for 8× PA-IC_90_ (*in vitro* protein-adjusted 90% CAB inhibitory concentration), 0.664 μg/mL for 4× PA-IC_90_, and 0.166 μg/mL for 1× PA-IC_90_. BLQ indicates that the CAB concentration was below the limit of quantification (<0.025 μg/mL). Shaded areas indicate that the participant was on antiretroviral therapy. Ag/Ab, antigen/antibody; EFV, efavirenz; FTC, emtricitabine; ND, not detected; TDF, tenofovir disoproxil fumarate; WT, wild type (no RAMs detected); XTC, lamivudine or emtricitabine.

**(ii) Infections that occurred <6 months after the last injection with one or more delayed injections (DX cases).** Participant DX1 received 6 CAB injections before the first HIV-positive visit (Fig. 2). A seventh injection was given 87 days later at the first HIV-positive visit before the site became aware of the infection. Three injections were administered late (>70 days [range, 87 to 146 days]); the CAB concentration was below the limit of quantification (BLQ) (<0.025 μg/mL) after the longest delay (146 days) and was <4× PA-IC_90_ (0.495 μg/mL) at the first HIV-positive visit. The VL was 1,212,660 copies/mL at the first HIV-positive visit. The site detected the infection at the same visit.

Participant DX2 received 9 CAB injections before the first HIV-positive visit (Fig. 2). A 10th injection was given at the first HIV-positive visit before the site was aware of the infection. The first 9 injections were administered on time, but there was an interval of 168 days between the 9th and 10th injections. Steep postpeak declines in CAB concentrations and increased absorption were also observed in this participant. At the first HIV-positive visit, the CAB concentration was BLQ, and the VL was 73,380 copies/mL. The site detected the infection at the same visit.

Participant DX3 received 6 CAB injections before the first HIV-positive visit (Fig. 2). Five of the injections were administered on time. However, there was an interval of 74 days between the second and third injections and an interval of 168 days between the last injection and the first HIV-positive visit. At the first HIV-positive visit, the CAB concentration was 0.041 μg/mL, and the VL was 48,030 copies/mL. The site detected the infection at the same visit.

**FIG 3:**
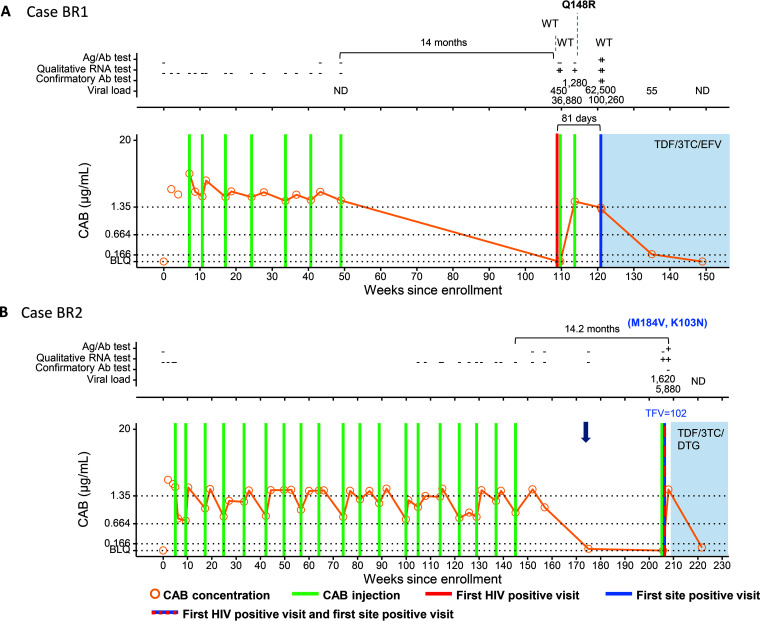
Summary of key events and laboratory results for participants in the cabotegravir (CAB) study arm of HPTN 083: cases BR1 (A) and BR2 (B). Results from testing performed at study sites are shown in File S2 in the supplemental material. Test results and key study events are shown as a function of the number of weeks from study enrollment based on calendar dates. Results obtained from testing performed at the HPTN Laboratory Center are shown above each graph. Positive and reactive results are indicated with a plus sign; negative and nonreactive results are indicated with a minus sign. IND indicates an indeterminate test result. VL values are shown (number of HIV RNA copies per milliliter). Results from HIV drug resistance testing are shown. All drug resistance mutations are shown for INSTIs; major INSTI mutations are shown in boldface type. Major drug resistance mutations for other drug classes are shown in blue text in parentheses. The brackets at the top of the graph show the number of days between the last injection and the first HIV-positive visit. Lower brackets show the number of days between the first site-positive visit and the first HIV-positive visit for cases where there was a delay in site-detection. The graphs show plasma CAB concentrations and key events. The key at the bottom describes the symbols used in the graphs. Horizontal lines indicate the following concentration cutoffs: 1.33 μg/mL for 8× PA-IC_90_ (*in vitro* protein-adjusted 90% CAB inhibitory concentration), 0.664 μg/mL for 4× PA-IC_90_, and 0.166 μg/mL for 1× PA-IC_90_. BLQ indicates that the CAB concentration was below the limit of quantification (<0.025 μg/mL). Shaded areas indicate that the participant was on antiretroviral therapy. The blue arrow indicates that tenofovir disoproxil fumarate-emtricitabine (TDF-FTC) was dispensed for preexposure prophylaxis. TFV indicates the concentration of tenofovir in plasma (nanograms per milliliter). 3TC, lamivudine; Ag/Ab, antigen/antibody; DTG, dolutegravir; EFV, efavirenz; ND, not detected; WT, wild type (no RAMs detected).

**(iii) Infections that occurred >6 months after the last injection with injections restarted on or after the first HIV-positive visit (BR cases).** Participant BR1 had 7 on-time injections and then had no study visits until the first HIV-positive visit 14 months later (Fig. 3). The CAB concentration at the first HIV-positive visit was BLQ. The site did not detect the infection at that visit, and the participant received an eighth injection on the same day and a ninth injection 28 days later. The VL was 450 copies/mL at the first HIV-positive visit. The site detected the infection 81 days later. In this case, the major INSTI RAM Q148R was detected 31 days after the first HIV-positive visit.

Participant BR2 completed 19 planned injections and then transitioned to daily oral TDF-FTC while awaiting the implementation of the OLE (Fig. 3). TDF-FTC was dispensed once, 210 days after the 19th CAB injection. The participant transitioned to the OLE 14.2 months after the 19th injection, which was also the first HIV-positive visit. The plasma tenofovir (TFV) concentration was 102 ng/mL at that visit; the CAB concentration was BLQ. The HIV rapid test was nonreactive at that visit, and the participant received a CAB injection on that date. The site became aware of the infection after the injection when results from local laboratory-based testing were available. This participant had dual-class resistance (K103N and M184V) at the first HIV-positive visit. It was not possible to determine whether the M184V mutation was transmitted or acquired from TDF-FTC exposure.

**(iv) Infections that occurred with no recent CAB administration (B cases).** Eleven cases had no CAB administration in the 6 months prior to the first HIV-positive visit (File S4). Two participants received no CAB injections (B6 and B7). The other 9 participants received a median of 9 CAB injections (range, 1 to 19 injections). The interval between the last injection and the first HIV-positive visit ranged from 210 to 950 days. In 9 cases, the CAB concentration at the first HIV-positive visit was BLQ; the remaining 2 cases had low but quantifiable CAB concentrations (B10, 0.067 μg/mL, 210 days after the last injection; B15, 0.110 μg/mL, 292 days after the last injection). In four cases, participants transitioned to TDF-FTC before the first HIV-positive visit. The plasma TFV concentration at the first HIV-positive visit was 109 ng/mL in one case (B13) and BLQ in three cases (B14 to B16). None of the 11 B cases had INSTI resistance.

**Delayed detection of HIV infection.** We evaluated the delays in the detection of HIV infection in 32/34 CAB arm cases identified to date (Tables 2 and 3). Two cases were excluded from the analysis since the participants were on antiretroviral treatment at the first HIV-positive visit where a sample was available for testing (B4 and B12). The 32 cases included 18 participants who received CAB within 6 months of the first HIV-positive visit (Table 2, group 1, A, C, D, DX, and BR cases) and 14 participants with no CAB administration in this period (Table 3, group 2, B cases). Detection of infection was delayed at the study sites in 15/32 cases (14/18 group 1 cases and 1/14 group 2 cases [*P* < 0.001]); the study drug was administered after the first HIV-positive visit in 13 of the 15 cases. HIV RNA screening using a sensitive assay (LLOD of 40 copies/mL) would have detected infection at the first HIV-positive visit in 12 of the 15 cases. The other three cases were detected using a qualitative RNA assay (LLOD of 30 copies/mL) but had negative results using a VL assay (lower limit of quantification [LLOQ] of 40 copies/mL); the VLs obtained for these samples using a single-copy RNA assay were 6.1, 7.4, and 15.3 copies/mL.

**TABLE 2:** Key laboratory results (CAB arm, group 1)^*a*^

Case ID	Subtype	No. of injections	No. of late injections	Time since last injection (days)	VL (copies/mL) at 1st positive visit	[CAB] (μg/mL) at 1st positive visit	DX delay	Time to site detection (days)	Drug administration after infection	Ag/Ab lab test result at 1st positive visit	Confirmatory Ab test result at 1st positive visit	Major INSTI RAM at 1st positive visit	Major INSTI RAM at any visit^*b*^	TDF-FTC administration	[TFV] (ng/mL) at 1st positive visit
A1	B	0	0	NA	4,010	BLQ	Yes	28	Yes	NR	NA	No	No	No	
A2	C	1	0	NA	50,080	BLQ	Yes	60	Yes	NR	NA	No	Yes	No	
A3	B	2	0	NA	1,360	BLQ	Yes	72	Yes	NR	NA	No	No	No	
A4	B	2	0	NA	44,180	BLQ	Yes	63	Yes	R	NEG	No	No	No	
C1	B	2	0	NA	120	6,301	Yes	47	Yes	NR	NA	No	Yes	No	
C2	BF	0	0	NA	494	BLQ	Yes	185	No	NR	NA	No	No	No	
C3	B	1	0	NA	SCA, 15.3	10.690	Yes	35	Yes	NR	NA	No	Yes	No	
D1	Likely B	10	1^*c*^	56	130	1.613	Yes	112	Yes	NR	NA	Yes	Yes	No	
D2	Likely B	6	0	14	SCA, 6.1	1.405	Yes	98	Yes	NR	NA	Failed testing	Yes	No	
D3	BF	5	0	56	860	1.504	Yes	117	Yes	NR	NA	No	Yes	No	
D4	C	4	0	13	<40	2.017	Yes	45	Yes	NR	NA	Failed testing	Yes	No	
D5	F	5	0	21	59	1.906	Yes	42	No	NR	NA	Yes	Yes	No	
D6	AE	15	0	53	2,020	1.824	Yes	59	Yes	NR	NA	No	Yes	No	
DX1	B	7	3	87	1,212,660	0.495	No		Yes	R	IND	No	NA (ART)	No	
DX2	BF	10	1	168	73,380	BLQ	No		Yes	R	IND	No	No	No	
DX3	B	6	1	169	48,030	0.041	No		No	R	POS	No	NA (ART)	No	
BR1	BC	9	1^*d*^	422	450	BLQ	Yes	81	Yes	NR	NA	No	Yes	No	
BR2	B	20	2^*d*^	425	1,620	BLQ	No		Yes	NR	NA	No	No	Yes	102

a Key laboratory results for cases in the cabotegravir (CAB) arm of HPTN 083 where the participant received CAB within 6 months of the first HIV-positive visit (group 1). The HIV subtype was determined by phylogenetic analysis of HIV sequences; if HIV sequences were not available, the likely subtype was assigned based on the predominant subtype in the region. The total number of injections received and the number of delayed injections (late, >2 weeks after the planned injection [>44 days after the 1st injection or >70 days after subsequent injections]) are shown. The number of days between the last injection and the first HIV-positive visit is shown. Some participants switched from CAB PrEP to TDF-FTC PrEP; for those participants, the concentration of tenofovir at the first HIV-positive visit is shown. Ab, antibody; Ag, antigen; ART, antiretroviral therapy; BLQ, below the limit of quantification; [CAB], CAB concentration (micrograms per milliliter); Case ID, case identifier; DX delay, delayed detection of HIV infection at the study site; FTC, emtricitabine; IND, indeterminate; INSTI, integrase strand transfer inhibitor; NA, not applicable (testing not indicated); NEG, negative; NR, nonreactive; POS, positive; RAM, resistance-associated mutation; R, reactive; TDF, tenofovir disoproxil fumarate; [TFV], tenofovir concentration (nanograms per milliliter); VL, viral load (HIV RNA copies per milliliter).

b The INSTI RAMs detected in each case are shown in File S5 in the supplemental material.

c This case was classified as a D case in previous publications. This participant had one delayed injection (the 6th of 9 injections was 75 days after the previous injection) and would be classified as a DX case under the updated classification system used in this report.

d In both of the BR cases, CAB injections were restarted before the site was aware that the participants had HIV infection. For case BR1, CAB-LA was restarted 422 days after a series of 7 on-time injections; the 8th injection was given 3 days after the first HIV-positive visit. For case BR2, CAB-LA was restarted in the open-label extension (OLE) 425 days after the participant completed all 19 scheduled injections in the main HPTN 083 study. In this case, the 20th injection was given at the first HIV-positive visit; one of the 19 previous injections was delayed (time interval, 75 days). Note that this report does not include participants who acquired HIV infection after the first OLE visit.

**TABLE 3:** Key laboratory results (CAB arm, group 2)^*a*^

Case ID^*b*^	Subtype	No. of injections	No. of late injections	Time since last injection (days)	VL (copies/mL) at 1st positive visit	[CAB] (μg/mL) at 1st positive visit	DX delay	Time to site detection (days)	Drug administration after infection	Ag/Ab lab test result at 1st positive visit	Confirmatory Ab test result at 1st positive visit	Major INSTI RAM at 1st positive visit	Major INSTI RAM at any visit	TDF-FTC administration	[TFV] (ng/mL) at 1st positive visit
B1	B	2	1	849	65,530	0.065	No		No	R	POS	No	NA (ART)	Yes	BLQ
B2	AB	0	0	NA	53,220	BLQ	No		No	R	POS	No	NA (ART)	No	
B3	AE	4	0	217	50,440	0.100	No		No	R	IND	No	NA (ART)	No	
B5	B	0	0	NA	2,559	BLQ	No		No	R	POS	No	NA (ART)	No	
B6	B	0	0	NA	28,040	BLQ	No		No	R	POS	No	No	No	
B7	B	0	0	NA	31,020	BLQ	No		No	R	POS	No	No	No	
B8	B	3	0	429	631,510	BLQ	No		No	R	POS	No	NA	No	
B9	B	6	0	315	1,387,280	BLQ	No		No	R	POS	No	No	No	
B10	AE	9	1	210	154,820	0.067	No		No	R	NEG	No	No	No	
B11	B	5	2	336	43,720	BLQ	No		No	R	POS	No	No	No	
B13	B	6	2	227	21,290	BLQ	No		No	R	POS	No	NA (ART)	Yes	109
B14	B	19	0	234	139,970	BLQ	No		No	R	POS	No	No	Yes	BLQ
B15	B	19	0	292	SCA, 7.4	0.110	Yes	85	Yes, TDF	NR	NA	Failed testing	No	Yes	BLQ
B16	B	19	1	324	167,840	BLQ	No		Yes, TDF	R	NEG	No	No	Yes	BLQ

a Key laboratory results for cases in the cabotegravir (CAB) arm of HPTN 083 where the participant had no CAB administration within 6 months of the first HIV-positive visit (group 2, no recent CAB administration). The HIV subtype was determined by phylogenetic analysis of HIV sequences. The total number of injections received and the number of delayed injections (late, >2 weeks after the planned injection [>44 days after the 1st injection or >70 days after subsequent injections]) are shown. The number of days between the last injection and the first HIV-positive visit is shown. Some participants switched from CAB PrEP to TDF-FTC PrEP; for those participants, the concentration of tenofovir at the first HIV-positive visit is shown. Ab, antibody; Ag, antigen; BLQ, below the limit of quantification; [CAB], CAB concentration (micrograms per milliliter); Case ID, case identifier; DX delay, delayed detection of HIV infection at the study site; IND, indeterminate; INSTI, integrase strand transfer inhibitor; NA, not applicable (testing not indicated); NEG, negative; NR, nonreactive; POS; positive; RAM, resistance-associated mutation; R, reactive; [TFV], tenofovir concentration (nanograms per milliliter); VL, viral load (HIV RNA copies per milliliter).

b Two B cases are not included in group 2 (B4 and B12) since the first HIV-positive visit in these cases occurred after the participants started antiretroviral therapy (ART). Case B4 started ART with tenofovir alafenamide, emtricitabine (FTC), and bictegravir 10 days prior to the first HIV-positive visit where a sample was available for testing. HIV genotyping failed at that visit, and no resistance results are available. Case B12 started ART with tenofovir disoproxil fumarate (TDF), emtricitabine, and efavirenz 74 days prior to the first HIV-positive visit where a sample was available for testing. No major resistance mutations were detected at that visit.

Three of the four cases in group 1 that did not have delayed detection were the cases with delayed injections (DX1 to DX3); in these cases, the interval between the last injection and the first HIV-positive visit ranged from 87 to 169 days. In the fourth case (BR2), HIV RNA screening was performed at the first HIV-positive visit as part of the OLE enrollment visit, 425 days after the last injection. Only one participant with no recent CAB administration had delayed detection of infection (B15). In this case, the participant had acute HIV infection with a VL of 7.4 copies/mL at the first HIV-positive visit; the infection was detected by the study site at the next visit.

### HIV drug resistance.

As noted above, major INSTI RAMs were detected in 3/18 new CAB cases: 2 with on-time injections (D5 and D6) and 1 where CAB was restarted after infection (BR1) (Files S5 and S6). In addition, two participants had a major nonnucleoside reverse transcriptase inhibitor (NNRTI) RAM (one with K103N [B11] and one with E138E/A [DX2]), one had a major protease inhibitor (PI) RAM (M46L [DX1]), and one had dual-class resistance (with M184V and K103N [BR2]).

We also evaluated the frequency of INSTI RAMs among the full set of 32 CAB cases noted above (Tables 2 and 3; File S5). We detected major INSTI RAMs in 10/32 cases. All 10 participants received CAB within 6 months of the first HIV-positive visit (group 1). The frequency of INSTI resistance was significantly higher among participants with recent CAB administration than among those with no recent CAB administration (10/18 in group 1 versus 0/14 in group 2 [*P* = 0.001]).

Eight of the 10 cases with major INSTI RAMs had a genotyping result from the first HIV-positive visit. In 6 of the 8 cases, HIV RNA screening would have detected the infection before major INSTI RAMs emerged. In one of the two cases where a major INSTI RAM was already present at the first HIV-positive visit, VL screening would have detected the infection before additional major INSTI RAMs accumulated.

Resistance results for cases in the TDF-FTC arm are described in File S2.

## DISCUSSION

This report provides new information on infections in cisgender men and TGW receiving CAB-LA for HIV PrEP, including participants with delayed injections, participants who discontinued CAB (“tail-phase” infections), and participants with undiagnosed infection who restarted CAB >6 months after the last injection. This report provides new insights into CAB-LA pharmacokinetics, the impact of CAB-LA on the detection of HIV infection, and the emergence of INSTI resistance in the setting of CAB-LA PrEP.

CAB-LA is delivered intramuscularly as a nanosuspension and exhibits “flip-flop” kinetics in which the terminal phase of the concentration-time profile is primarily attributable to drug absorption rather than drug elimination (8, 9). Individuals with increased absorption may exhibit steeper declines between peak and trough CAB concentrations. The CAB-LA regimen used in this study was targeted to achieve concentrations of ≥4× PA-IC_90_ in 80% of individuals and ≥8× PA-IC_90_ in 50% of individuals. Pharmacologic variability was observed in participants who received on-time injections. In case D5, CAB appeared to be absorbed more quickly following each CAB injection than population-based values; similar postpeak declines in CAB concentrations were observed in two other cases (DX2 and B11). In case D6, trough CAB concentrations of <8× PA-IC_90_ were noted in 5/6 events prior to the first HIV-positive visit. The remaining 16/18 new cases in the CAB arm occurred when CAB injections were delayed or discontinued. In 4/6 cases where participants switched from CAB-LA to TDF-FTC PrEP, TFV concentrations at the first HIV-positive visit indicated poor TDF-FTC adherence. These findings highlight challenges associated with the interruption of CAB-LA due to limited drug access or other issues.

This study and our previous reports (3, 10) demonstrate that most infections in persons using CAB-LA PrEP would have been detected earlier with HIV RNA screening. However, in some cases, the level of HIV RNA at the first HIV-positive visit was below the level of detection of most commercial assays. The guidelines in the prescribing information for Apretude (brand name of CAB-LA) (11) and recent guidelines from the U.S. CDC (12) include HIV RNA screening for persons receiving CAB-LA PrEP. The CDC guidelines also recommend testing with an HIV RNA assay every 3 months for a year after the last CAB injection. We previously demonstrated that the detection of HIV infections in persons receiving CAB-LA is often delayed using HIV diagnostic algorithms that do not include an HIV RNA test. Here, we demonstrate that detection delays usually occur in cases where the first HIV-positive visit is <6 months after the last CAB injection. This finding is based on analysis of HIV infections in cisgender men and TGW. The period of delayed detection of HIV infection may be longer in cisgender women since the apparent terminal half-life of CAB-LA is longer in persons assigned female at birth (13).

One consequence of the delayed detection of infection in the setting of CAB-LA PrEP is the emergence of INSTI resistance (4). We find that INSTI resistance can emerge quickly following CAB-LA exposure, even when CAB concentrations are ≥8× PA-IC_90_ and the HIV VL is low. To date, all cases with major INSTI RAMs occurred in HPTN 083 participants who received CAB within 6 months of the first HIV-positive visit. This period may be longer in persons assigned female at birth because of sex-based differences in the terminal half-life of CAB-LA.

In HPTN 083, HIV RNA screening would have detected most HIV infections before major INSTI RAMs were present (4). In one case, HIV RNA testing detected an infection at the first OLE visit. However, because the rapid HIV test was nonreactive, the participant received a CAB injection before the site was aware of the infection. The site became aware of the infection when the HIV RNA result became available, before INSTI resistance emerged. In another case, CAB-LA was restarted after missed study visits while the participant was still in the parent HPTN 083 study; HIV RNA screening was not routinely performed in this phase of the trial. This participant received two CAB injections before the infection was detected and had INSTI resistance at the first site-positive visit. These two cases provide support for HIV RNA screening before restarting CAB-LA injections in persons with delayed injections and in persons who are restarting CAB-LA after discontinuing the drug.

While there are some benefits of HIV RNA screening in the setting of CAB-LA PrEP, this testing is not feasible in some settings and adds considerable cost and complexity for CAB-LA PrEP implementation. Given the potent and long-lasting suppressive effect of CAB-LA, it may also be difficult to distinguish false-positive and true-positive HIV RNA results, especially when RNA is detected at low levels. The World Health Organization guidelines for HIV testing in the setting of CAB-LA PrEP do not include HIV RNA screening (14). More research is needed to evaluate the advantages and disadvantages of HIV RNA screening in this setting.

Analysis of HIV infections is ongoing in HPTN 083, HPTN 084, and the OLE phases of both trials. Results from those studies will provide more information on infections that occur during the CAB tail phase and infections in the setting of the reinitiation of CAB-LA after delayed or discontinued injections. Those studies will also provide more information about the use of HIV RNA screening in the setting of CAB-LA PrEP, which will help identify optimal HIV diagnostic algorithms for use in this setting.

## MATERIALS AND METHODS

### Study cohort.

The design and outcomes of the blind phase of HPTN 083 (ClinicalTrials.gov identifier NCT02720094) were described previously (1). Participants in the CAB arm received daily oral CAB for 5 weeks followed by 600-mg CAB injections for up to 3 years (two injections 1 month apart followed by injections every 2 months). Injections were classified as delayed if they occurred >2 weeks after the planned injection date. Participants who stopped injections early or completed 19 scheduled injections were offered 48 weeks of daily oral TDF-FTC PrEP. Participants in the TDF-FTC arm received daily oral TDF-FTC. The blind phase of the trial was stopped early by an independent data safety monitoring board on 14 May 2020. Participants were unblinded and continued their original randomized study regimen while awaiting the implementation of the OLE.

HIV testing performed at study sites and the HPTN Laboratory Center was used to determine HIV infection status and identify the first HIV-positive visit. This report includes HIV infections that occurred through 15 May 2021 that were detected by the sites through 15 November 2021; this provided a 6-month window after the end of the first unblinded year for the sites to detect infections. Data are included for study visits through 15 November 2021. Cases were not included if the first HIV-positive visit was after the first visit in the OLE.

### Testing at study sites.

Participants were screened for HIV infection at each study visit using one or two HIV rapid tests and an FDA-cleared laboratory-based antigen/antibody (Ag/Ab) test. In the OLE, HIV RNA testing was performed at every injection visit using an assay with an LLOD of 50 copies/mL or lower. HIV infection was confirmed using locally available tests. In selected cases, an ultrasensitive HIV DNA assay was performed in real time at Johns Hopkins University (Baltimore, MD, USA) (3). The first site-positive visit was defined as the first visit near the time of confirmed infection when the site obtained a reactive or positive test result.

### Retrospective testing.

Retrospective testing was performed at the HPTN Laboratory Center to confirm HIV infection and determine the timing of infection (see File S1 in the supplemental material) (3). This testing included qualitative RNA testing, VL testing, HIV genotyping, and measurements of drug concentrations (3). Qualitative RNA testing was performed using the Aptima HIV-1 RNA qualitative assay (Hologic, Inc., San Diego, CA). VL testing was performed using the RealTime HIV-1 viral load assay (Abbott Molecular, Des Plaines, IL) (LLOQ of 40 copies/mL); selected samples were analyzed using a single-copy RNA assay (3). Drug concentrations were determined by liquid chromatography-tandem mass spectrometry (LC-MS/MS) (3, 15, 16). HIV genotyping was performed using the GenoSure PRIme assay (Monogram Biosciences, South San Francisco, CA) for samples with VLs of ≥500 copies/mL. Samples with VLs of <500 copies/mL were analyzed using a low-VL single-genome sequencing INSTI genotyping assay (low-VL SGS-IN) (CAB arm only) (4). Resistance-associated mutations (RAMs) were classified as major or accessory mutations using the Stanford University HIV Drug Resistance Database (17, 18). HIV subtyping was performed at Monogram Biosciences (3).

### Interpretation of study drug concentrations.

In the TDF-FTC arm, adherence to oral TDF-FTC was assessed using tenofovir (TFV) concentrations in plasma and tenofovir diphosphate (TFV-DP) concentrations in dried blood spots (DBSs) (3). In the CAB arm, some participants transitioned to oral TDF-FTC after receiving CAB-LA injections; in these cases, TDF-FTC adherence was assessed based on plasma TFV concentrations only due to infrequent DBS storage. The *in vitro* protein-adjusted 90% CAB inhibitory concentration (PA-IC_90_) is 0.166 μg/mL (19, 20). CAB concentrations were stratified into four groups relative to the PA-IC_90_: <1× PA-IC_90_, 1× to <4× PA-IC_90_ (0.166 to 0.664 μg/mL), 4× to <8× PA-IC_90_ (0.664 to 1.33 μg/mL), and ≥8× PA-IC_90_ (≥1.33 μg/mL).

### Ethical considerations.

The HPTN 083 protocol was approved by the institutional review boards and/or ethics committees and ministries of health for all participating sites (1). All participants provided written informed consent.
