# Supplementary material for: Extended Analysis of HIV Infection in Cisgender Men and Transgender Women Who Have Sex with Men Receiving Injectable Cabotegravir for HIV Prevention: HPTN 083
Source: Antimicrob Agents Chemother. 2023 Mar 30;67(4):e00053-23. doi: 10.1128/aac.00053-23 (PMC10112247; doi:10.1128/aac.00053-23)
Supplement: Supplemental file 1 — Supplemental material. Download aac.00053-23-s0001.pdf, PDF file, 0.5 MB [file aac.00053-23-s0001.pdf]

SUPPLEMENTARY FILES

Supplementary File 1	Retrospective virology testing	Pages 2-3
Supplementary File 2	Key laboratory results (TDF/FTC arm)	Pages 4-6
Supplementary File 3	Comparison of site and HPTN LC test results (D, DX, and BR cases)	Pages 7-10
Supplementary File 4	Cases with no recent CAB administration (CAB arm, B cases)	Pages 11-22
Supplementary File 5	INSTI resistance summary	Page 23
Supplementary File 6	Results from the low viral load INSTI genotyping assay	Page 24

<u>Supplementary File 1. Retrospective virology testing</u>

Retrospective testing was performed at the HPTN Laboratory Center for all cases where a reactive or positive HIV test result was obtained at the study site. Results from this testing were not returned to study sites or participants. Testing used to determine HIV infection status and the timing of infections events used a pre-planned testing algorithm with supplemental testing (Ref 1). The testing schema used in this report to determine HIV status and the timing of HIV infection is shown in the table below. Modifications from methods used in the blinded phase of the study are noted in italic text. HIV testing was not required after initiation of antiretroviral therapy.

<u>Table. Schema used for HIV testing at the HPTN Laboratory Center.</u>

**Assay**	**CAB arm**	**TDF/FTC arm**
Qualitative RNA test	• Enrollment<br>• *Weeks 2, 4, and 5*<br>• *First HIV positive visit*<br>• *All visits in the two years prior to the first HIV positive visit or three visits prior to the first HIV positive visit, whichever is greater*<br>• *All visits between the first HIV positive visit and the second visit where the confirmatory Ab test is positive*	• Enrollment<br>• Weeks 2, 4, and 5<br>• First site positive visit; continue testing prior visits until a non-reactive result is obtained<br>• All subsequent visits until the second visit where the confirmatory Ab test is positive
Ag/Ab test	• Enrollment<br>• *One visit* prior to the first visit where a reactive qualitative RNA result was obtained<br>• First visit where a reactive qualitative RNA result was obtained<br>• All subsequent visits until the second visit where the confirmatory Ab test is positive	Same as for the CAB arm
Confirmatory Ab test	• All visits where the Ag/Ab test was reactive	Same as for the CAB arm
Single copy RNA test	• First HIV positive visit, if the qualitative RNA test is reactive, and there are no other reactive/positive results from other tests performed at the HPTN Laboratory Center	Same as for the CAB arm

<u>Tests used</u>: Qualitative RNA test: Aptima HIV-1 RNA Qualitative Assay; Ag/Ab test: Architect HIV Ag/Ab Combo assay; confirmatory Ab test: Geenius HIV 1/2 Supplemental Assay; single copy RNA test (laboratory-developed test).

Abbreviations: Ab: antibody; Ag; antigen; CAB: cabotegravir; HPTN: HIV Prevention Trials Network; TDF/FTC: tenofovir disoproxil fumarate.

Adjudication of test results

HIV test results from study sites and the HPTN Laboratory Center were reviewed by an independent Endpoint Adjudication Committee for all cases where at least one reactive or positive HIV test was obtained at the study site. The adjudication committee made a final determination of HIV status and identified the first HIV positive visit based on the available data. Confirmation

of HIV infection by the adjudication committee required a positive RNA test, a positive confirmatory Ab test, or a positive HIV DNA test with a result above the lower limit of detection.

Classification of study visits

The first site positive visit is defined as the first visit near the time of confirmed HIV infection where the site obtained a reactive or positive HIV test result. In some cases, a reactive HIV test was obtained earlier in the study with no other reactive/positive tests near the time of that visit; those results are not used to determine the first site HIV positive test.

The first HIV positive visit is defined as the first visit with evidence of HIV infection, as determined by the HPTN 083 Endpoint Adjudication Committee. This determination was based on review of results from testing performed at study sites and the HPTN Laboratory Center. In some cases, data from the HPTN Laboratory Center was not available for the primary review by the adjudication committee. Results from the single copy RNA test were used to confirm infection at the first HIV positive visit if the qualitative RNA result was the only other reactive/positive HIV test; however, this test was not used alone to determine the date of the first HIV positive visit.

Further characterization of HIV infections

Additional testing was used to characterize infections. This included HIV viral load testing, drug resistance testing and measurement of drug concentrations. The assays used for this testing are noted in the Methods section. Additional details are provided in previous reports (Low VL SGS-IN assay: Ref 2; all other assays: Ref 1).

References:

(1) Marzinke MA, Grinsztejn B, Fogel JM, Piwowar-Manning E, Li M, Weng L, et al. Characterization of human immunodeficiency virus (HIV) infection in cisgender men and transgender women who have sex with men receiving injectable cabotegravir for HIV prevention: HPTN 083. J Infect Dis. 2021;224(9):1581-92.

(2) Eshleman SH, Fogel JM, Halvas EK, Piwowar-Manning E, Marzinke MA, Kofron R, et al. HIV RNA screening reduces integrase strand transfer inhibitor resistance risk in persons receiving long-acting cabotegravir for HIV. J Infect Dis. 2022; In Press.

Supplementary File 2. Key laboratory results (TDF/FTC arm)

Thirty-four additional TDF/FTC infections were identified in this study (E43-E76). Two of the 34 infections occurred in the blinded phase of the study (E43, E44) and 32 occurred in the first unblinded year (E45-E76); two cases in the unblinded year could not be fully assessed since the participants were already on ART (E75 had no sample stored at the first HIV positive visit and started ART before the next visit; E76 was on ART at the first HIV positive visit). Characteristics of these infections are shown in the table below.

Adherence to oral TDF/FTC was assessed based on concentrations of tenofovir in plasma and tenofovir-diphosphate in dried blood spot samples (Ref 1). Data are shown for the first HIV positive visit; the number of days between enrollment and this visit is shown (Days since enroll). Detection delay indicates the number of days between the first HIV positive visit and the visit where the site first detected the possibility of infection (first site positive visit). Results of HIV tests and HIV genotyping are shown for the first HIV positive visit. Major resistance mutations are shown in bold text.

TFV and TFV-DP concentrations were consistent with daily TDF/FTC use in two cases (E57, E59) and with partial adherence in two cases (E64, E67); the other 30 cases had poor adherence. Detection of HIV infection was delayed at study sites in three cases (E43, E65 and E66). All three cases had acute (RNA only) infection at the first HIV positive visit; all three infections were detected at the study sites at the following visit (14-58 days later). Two of the three cases had a positive Geenius result at the first site positive visit; the third case had an indeterminate Geenius result at that visit with a positive result at the following visit 67 days later.

HIV genotyping was performed at the first HIV positive visit for the 27 of the 34 cases (three had viral loads <500 copies/mL; one had insufficient plasma available for testing; two were on ART; one was tested at the next visit). Results were obtained for 26 cases (one failed testing). Major RAMs were detected in ten cases. Six cases had the major NRTI RAM, M184I/V, at the first HIV positive visit; one of those cases also had the K65R mutation. These mutations are associated with resistance to TDF/FTC. In addition, four of the six cases also had one or two major NNRTI RAMs; one of those four cases also had the PI RAM, M46L. The remaining four cases with a major RAM detected all had a single NNRTI mutation detected. No other major PI RAMs were detected and no major INSTI RAMs were detected.

The table below shows characteristics of the 30 of the 32 TDF/FTC cases described in this report (E43-E74); this analysis could not be performed for two cases (E75 and E76) since the participant was on antiretroviral therapy at the first HIV-positive visit where a sample was available for testing. Data are shown for the first HIV positive visit; the number of days between enrollment and this visit is shown (Days since enroll). Adherence to oral TDF/FTC was assessed based on concentrations of tenofovir in plasma and tenofovir-diphosphate in dried blood spot samples (Ref 1). Detection delay indicates the number of days between the first HIV positive visit and the visit where the site first detected the possibility of infection (first site positive visit). Results of HIV tests and HIV genotyping are shown for the first HIV positive visit. Major resistance mutations are shown in bold text.

Reference:

(1) Marzinke MA, Grinsztejn B, Fogel JM, Piwowar-Manning E, Li M, Weng L, et al. Characterization of human immunodeficiency virus (HIV) infection in cisgender men and transgender women who have sex with men receiving injectable cabotegravir for HIV prevention: HPTN 083. J Infect Dis. 2021;224:1581-92.

Case ID	HIV subtype	TDF/FTC adherent	Days since enroll	Detection delay	1st HIV positive visit							
					Viral load (c/mL)	Ag/Ab test	Confirmatory Ab test	Any major RAM	Drug resistance mutations detected			
									NRTI	NNRTI	PI	INSTI
E43	B	No	288	**Yes**	900	**NR**[a]	N/A	No		K103R		
E44	B	No	408	No	57,390	R	POS	No				M50I
E45	BF	No	330	No	221,220	R	POS	**Yes**	**M184V/I**		K20M, A71T	
E46	Complex	No	345	No	85,850	R	POS	**Yes**		**K103N**		
E47	B	No	430	No	204,100	R	POS	No			L10V	
E48	BC	No	523	No	14,200	R	POS	**Yes**	K219R	**V106I**	L10I, A71T	
E49	B	No	524	No	17,140	R	POS	**Yes**	**M184I/V**	**E138A**, V179I	**M46M/L,** A71V	M50I
E50	N/D	No	553	No	9,620	R	POS	Failed testing				
E51	B	No	591	No	7,310	R	POS	No				
E52	B	No	595	No	2,796,320	R	POS	No				M50I
E53	BF	No	641	No	142,570	R	POS	No			K20M	
E54	B	No	660	No	3,445,350	R	**IND**[b]	No				
E55	BF	No	682	No	3,611,120	R	POS	No			K20M	
E56	B	No	715	No	511,980	R	POS	No			L10V, A71V	L74I
E57	N/D	**Yes**	721	No	242	R	POS	Not tested (VL<500)				
E58	B	No	722	No	221,460	R	POS	No		V179D	L10I, A71T	
E59	B	**Yes**	734	No	650	R	POS	**Yes**	**M184V**	K103R, V179D, **Y188L**	L10I, A71T	L74I
E60	B	No	743	No	31,732,440	R	**IND**[c]	No				
E61	B	No	751	No	43,580	R	POS	No				M50I, V151I
E62	B	No	754	No	48,680	R	POS	**Yes**		**K103S**		
E63	B	No	807	No	18,380	R	POS	**Yes**		**K103N**		
E64	AE	**Partial**	815	No	14,400	R	POS	**Yes**	**M184I/V**		K20R	
E65	B	No	847	**Yes**	540	**NR**[d]	N/A	Not tested[d]				
E66	B	No	882	**Yes**	9.5 (SCA)	**NR**[e]	N/A	No				M50I
E67	N/D	**Partial**	885	No	SCA Neg	R	POS	Not tested (VL<500)				

E68	BF	No	901	No	162,930	R	**NEG**[f]	No			K20M, A71T	
E69	B	No	910	No	358,700	R	POS	**Yes**	**M184V**	K101Q, **K103N**, **E138A**, P225H		
E70	N/D	No	913	No	Not tested (QNS)[g]	R	POS	Not tested (QNS)				
E71	B	No	951	No	711,390	R	POS	No				M50I
E72	C	No	980	No	4,870	R	POS	**Yes**	**K65R**, K70T, V118I, **M184V**	**K103S**, **V106M**		M50I, L74I
E73	AE	No	987	No	1,233,600	R	POS	No		V179I	K20R	
E74	N/D	No	1010	No	358	R	POS	Not tested (VL<500)				
E75	This case could not be fully assessed since there was no sample available for testing after infection before antiretroviral therapy initiation.											
E76	This case could not be fully assessed since the participant was on antiretroviral therapy at the first HIV positive visit.											

Table footnotes:

[a] Case E43: The Ag/Ab test was reactive and the Ab discriminatory test was indeterminate at the next visit 14 days later; the Ab discriminatory test was positive at the next visit.

[b] Case E54: The Ab discriminatory test was positive 6 days later.

[c] Case E60: The Ab discriminatory test was positive 15 days later.

[d] Case E65: The Ag/Ab test was reactive and the Ab discriminatory test was positive at the next visit 58 days later. HIV genotyping was performed at the visit following the first HIV positive visit 58 days later; two accessory mutations were identified at that visit (PI: L10I and INSTI: T97A).

[e] Case E66: The Ag/Ab test was reactive and the Ab discriminatory test was positive at the next visit 45 days later.

[f] Case E68: The Ab discriminatory test was negative 7 days later; the participant started antiretroviral treatment after that visit (prior to the next follow-up visit).

[g] Case E70: Viral load testing could not be performed at the first HIV positive visit because there was not sufficient plasma for testing (QNS); the viral load was 1,503,360 copies/mL two days later.

Abbreviations: Ab: antibody; Ag/Ab: laboratory-based antigen/antibody test; c/mL: copies/milliliter; ID: case identifier; IND: indeterminate; INSTI: integrase strand transfer inhibitor; N/A: not applicable (testing not indicated); N/D: HIV subtype not determined (HIV genotyping not performed or failed); NEG: negative; NNRTI: non-nucleoside reverse transcriptase inhibitor; NR: non-reactive; NRTI: nucleoside/nucleotide reverse transcriptase inhibitor; PI: protease inhibitor; POS: positive; QNS: quantity not sufficient for testing; R; reactive; RAM: resistance associated mutation; SCA: single copy RNA assay; TDF/FTC: tenofovir disoproxil fumarate/emtricitabine.

Supplementary File 3. Comparison of site and HPTN LC test results (D, DX, and BR cases)

Legend for figures.

Each figure shows laboratory results for one case. Results are shown for real-time testing performed at study sites (Site Testing) and retrospective testing performed at the HPTN Laboratory Center (HPTN LC Testing). Study visits are shown on the left. A single asterisk indicates that a cabotegravir CAB injection was given at that visit. A double asterisk indicates that antiretroviral therapy (ART) was started at that visit or after that visit, before the next study visit. The first HIV positive visit (1st HIV pos) and first site positive visit (1st SITE pos) are noted. Viral load results are reported as HIV RNA copies/mL. HIV DNA results are reported as copies/million cells.

Abbreviations: Ab: antibody; Ag/Ab test: instrumented antigen/antibody test; ART: antiretroviral therapy; F/U: follow-up visit; IND: indeterminate; LLOD: lower limit of detection; ND: not determined/not tested; NEG: negative; NR: non-reactive; OLE: open-label extension study POS: positive; R: reactive. Antiretroviral drugs in treatment regimens are abbreviated as follows: 3TC: lamivudine; DRVr: ritonavir-boosted darunavir; DTG: dolutegravir; EFV: efavirenz; FTC: emtricitabine; TDF: tenofovir disoproxil fumarate; XTC: 3TC or FTC.

Case D5

			Site Testing				HPTN LC Testing			
	Visit type	Diagnosis visit type	Rapid test	Ag/Ab test	DNA test	Viral load	Ag/Ab test	Qualitative RNA test	Confirmatory Ab test	Viral load
42 days	Week 35	1st HIV pos	NR	NR			NR	R		59
	Week 41	1st SITE pos	R	R		16,194	R	R	IND	17,990
	Week 41**		R	R	Detect, <LLOD	526	R	R	POS	593
	Interim									ND
	F/U Week 12									ND
	F/U Week 24					ND	R	NR	POS	ND
	F/U Week 36									ND
	F/U Week 48					ND	R	R	POS	ND

**Started ART with TDF/3TC/DRVr

Case D6

			Site Testing			HPTN LC Testing			
	Visit type	Diagnosis visit type	Rapid test	Ag/Ab test	Viral load	Ag/Ab test	Qualitative RNA test	Confirmatory Ab test	Viral load
59 days	Week 113*	1st HIV pos	NR	NR		NR	R		2,020
	Week 121	1st SITE pos	R	R	17,200	R	R	POS	9,000
	Interim**		R	R	13,700	R	R	POS	13,380
	F/U Week 12								ND
	F/U Week 24				ND		NR		<40

**Started ART with TDF/3TC/EFV

Case DX1

Visit type	Diagnosis visit type	Site Testing: Rapid test	Site Testing: Ag/Ab test	Site Testing: Viral load	Site Testing: Confirmatory Ab test	HPTN LC Testing: Ag/Ab test	HPTN LC Testing: Qualitative RNA test	HPTN LC Testing: Confirmatory Ab test	HPTN LC Testing: Viral load
Week 65*	1st HIV/ SITE pos	NR	R	1,252,682		R	R	IND	1,212,660
Week 65**		R	R	251,494	IND	R	R	IND	202,620
F/U Week 12									ND
F/U Week 24				<40					<40
F/U Week 36									ND

**Started ART with TDF/FTC/EFV 24 days later.

Case DX2

Visit type	Diagnosis visit type	Site Testing: Rapid test	Site Testing: Ag/Ab test	Site Testing: Viral load	Site Testing: Confirmatory Ab test	HPTN LC Testing: Ag/Ab test	HPTN LC Testing: Qualitative RNA test	HPTN LC Testing: Confirmatory Ab test	HPTN LC Testing: Viral load
Week 81*	1st HIV/ SITE pos	NR	R	150,947	IND	R	R	IND	73,380
Interim**		NR	R	285,295	IND	R	R	NEG	113,620
F/U Week 12						R	R	IND	56
F/U Week 24				612		R	R	IND	550
F/U Week 36									<40
F/U Week 48				<40					ND

**Started ART with TDF/XTC/EFV 14 days later.

Case DX3

Visit type	Diagnosis visit type	Site Testing: Rapid test	Site Testing: Ag/Ab test	Site Testing: Viral load	Site Testing: Confirmatory Ab test	HPTN LC Testing: Ag/Ab test	HPTN LC Testing: Qualitative RNA test	HPTN LC Testing: Confirmatory Ab test	HPTN LC Testing: Viral load
Week 65	1st HIV/ SITE pos	R	R	79,325	POS	R	R	POS	48,030
Week 65**		R	R	57,311	POS	R	R	POS	42,270
F/U Week 12									430
F/U Week 24				70			R		<40
F/U Week 36									<40
F/U Week 48				<40					<40

**Started ART with TDF/XTC/EFV 9 days later.

Case BR1

	Visit type	Diagnosis visit type	Site Testing: Rapid test	Site Testing: Ag/Ab test	Site Testing: DNA test	Site Testing: Viral load	HPTN LC Testing: Ag/Ab test	HPTN LC Testing: Qualitative RNA test	HPTN LC Testing: Confirmatory Ab test	HPTN LC Testing: Viral load
	Interim after week 49	1st HIV pos	NR	NR			NR	R		450
81 days	Week 105*		NR	NR			NR	R		36,880
	Week 113*		NR	NR			NR	R		1,280
	Week 121	1st SITE pos	R	R		167,000	R	R	POS	62,500
	Interim**		R	R	Detect, >100	119,000	R	R	POS	100,260
	F/U Week 12									55
	F/U Week 24					55				ND

**Started ART with TDF/3TC/EFV

Case BR2

Visit type	Diagnosis visit type	Site Testing: Rapid test 1	Site Testing: Rapid test 2	Site Testing: Ag/Ab test	Site Testing: Viral load	Site Testing: Confirmatory Ab test	HPTN LC Testing: Ag/Ab test	HPTN LC Testing: Qualitative RNA test	HPTN LC Testing: Confirmatory Ab test	HPTN LC Testing: Viral load
OLE Day 0*	1st HIV/ SITE pos	NR	NR	NR	1,597		NR	R		1,620
Interim**		R	R	IND	5,364	IND	R	R	NEG	5,880
F/U Week 12										ND

**Started ART with TDF/3TC/DTG

Supplementary File 4. Cases with no recent CAB administration (CAB arm, B cases)

Eighteen new incident infections were identified in the CAB arm. Eleven of these cases had no CAB administration in the prior six months. In two cases, the participants received no CAB injections (B6 and B7). The remaining nine cases (B8-B16) included five cases where the participant was provided with daily oral TDF/FTC after discontinuing CAB injections (B6, B13-16). The figures below present key data from each of the 11 cases with no recent CAB administration.

Figure legend.
The figures provide a summary of key events and laboratory results for participants in the CAB study arm of HPTN 083 who had no recent CAB administration (B cases). Study visits are shown on the left. Tables show results for real-time testing performed at study sites (Site Testing) and retrospective testing performed at the HPTN Laboratory Center (HPTN LC Testing). A double asterisk indicates that antiretroviral therapy (ART) was started at that visit or after that visit, before the next study visit. The first HIV positive visit (1st HIV pos) and first site positive visit (1st SITE pos) are noted. Viral load results are reported as HIV RNA copies/mL. HIV DNA results are reported as copies/million cells. Graphs show results from the HPTN LC and key study events. The X axis indicates the number of weeks from study enrollment. Test results from the HPTN LC are shown above the graphs. Positive and reactive laboratory test results are indicated with a plus sign (+); negative and non-reactive results are indicated with a minus sign (-). IND indicates an indeterminate test result. Viral load values are reported as HIV RNA copies/mL; results noted as <40 indicate that HIV RNA was detected at a level below 40 copies/mL. Results from HIV drug resistance testing are shown. All drug resistance mutations are shown for INSTIs; major INSTI mutations are shown in bold text. Major drug resistance mutations for other drug classes are shown in blue text in parentheses. Brackets above the graphs show the number of days between the last CAB injection and the first site positive visit. The legend at the bottom of each figure describes symbols used in the graphs. CAB concentration is shown on the Y axis. Horizontal lines indicate the following CAB concentration cut-offs: 1.33 µg/mL = 8x PA-IC_{90}; 0.664 µg/mL = 4x PA-IC_{90}; 0.166 µg/mL = 1x PA-IC_{90}. BLQ indicates that the CAB concentration was below the limit of quantification (<0.025 µg/mL). Shaded areas indicate that the participant was on antiretroviral therapy. Blue arrows indicate that TDF/FTC was dispensed for pre-exposure prophylaxis. TFV indicates the concentration of tenofovir in plasma (ng/mL).

Abbreviations: Ab: antibody; Ag/Ab: instrumented antigen/antibody test; ART: antiretroviral therapy; BLQ: below the limit of quantification; CAB: cabotegravir; F/U: follow-up visit; IND: indeterminate; INSTI: integrase strand transfer inhibitor; LLOD: lower limit of detection; mL: milliliter; ND: not determined/not tested; NEG: negative; NNRTI: non-nucleoside reverse transcriptase inhibitor; NR: non-reactive; OLE: open-label extension study; PA-IC_{90}: *in vitro* protein-adjusted 90% CAB inhibitory concentration; POS: positive; R: reactive; SCA: single-copy RNA assay; µg: microgram; WT: wild type: no INSTI resistance associated mutations detected and no major mutations detected in another drug class. Antiretroviral drugs in treatment regimens are abbreviated as follows: ATVr: ritonavir-boosted atazanavir; BIC: biktarvy; COBI: cobicistat; DRV: darunavir; EFV: efavirenz; FTC: emtricitabine; LPVr: ritonavir-boosted lopinavir; TAF: tenofovir alafenamide; TDF: tenofovir disoproxil fumarate.

Case B6

Visit type	Diagnosis visit type	Site Testing: Rapid test 1	Site Testing: Rapid test 2	Site Testing: Ag/Ab test	Site Testing: Viral load	Site Testing: Confirmatory Ab test	HPTN LC Testing: Ag/Ab test	HPTN LC Testing: Qualitative RNA test	HPTN LC Testing: Confirmatory Ab test	HPTN LC Testing: Viral load	HPTN LC Testing: Resistance
Yearly Visit 1	1st HIV pos/ SITE pos	R	R	R	20,473	POS	R	R	POS	28,040	M50I
Interim**		R	R	R	61,163	POS	R	R	POS	41,150	M50I
Interim										<40	
Interim					<40			R		56	
Interim										50	
Interim					ND					<40	

**Started ART with TDF/FTC/EFV 39 days later, then switched to TDF/FTC/LPVr.

HIV subtype B

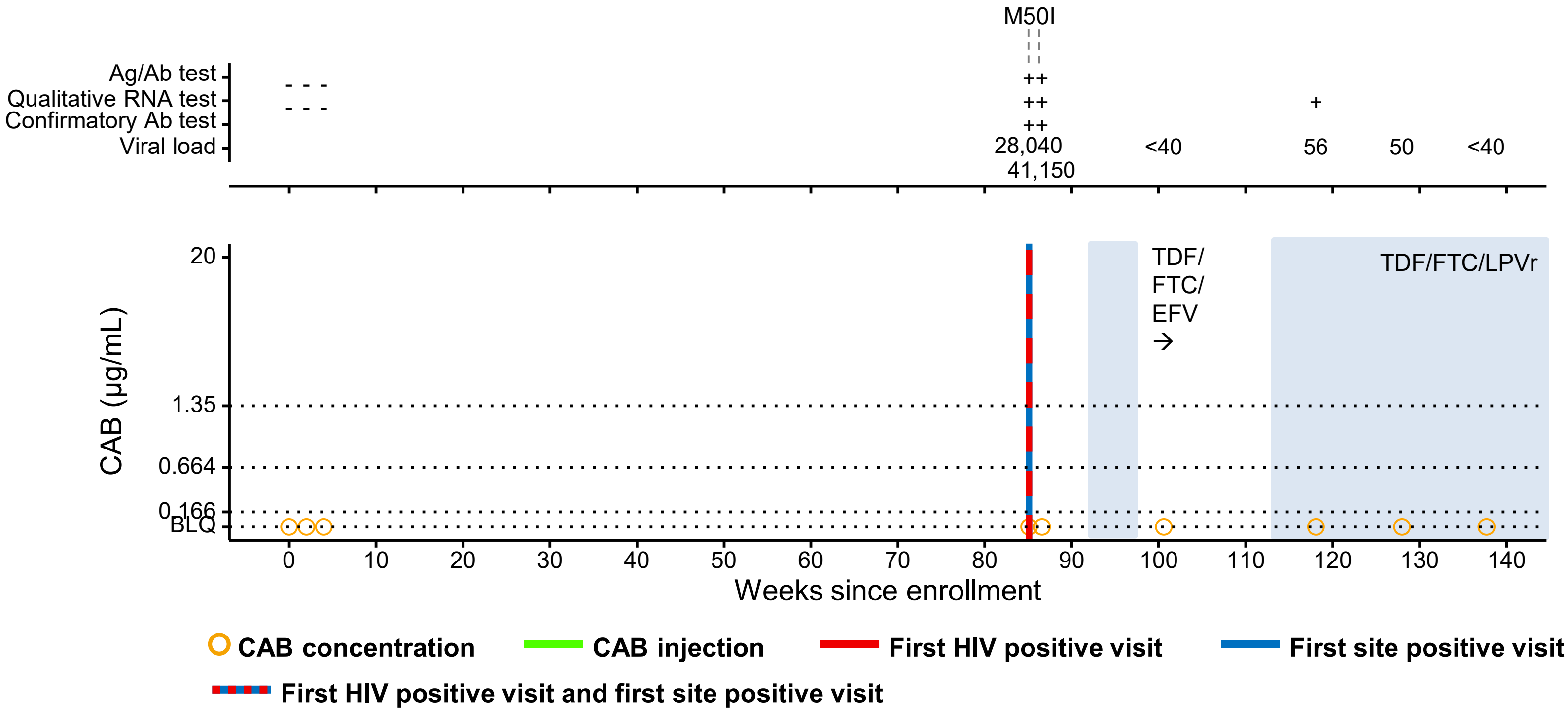

Case B7

		Site Testing					HPTN LC Testing				
Visit type	Diagnosis visit type	Rapid test 1	Rapid test 2	Ag/Ab test	Viral load	Confirmatory Ab test	Ag/Ab test	Qualitative RNA test	Confirmatory Ab test	Viral load	Resistance
Yearly visit 2	1st EAC/SITE pos	R	R	R	22,026	POS	R	R	POS	31,020	WT
Interim		R	R	R	3,271	POS	R	R	POS	5,690	WT

HIV subtype B

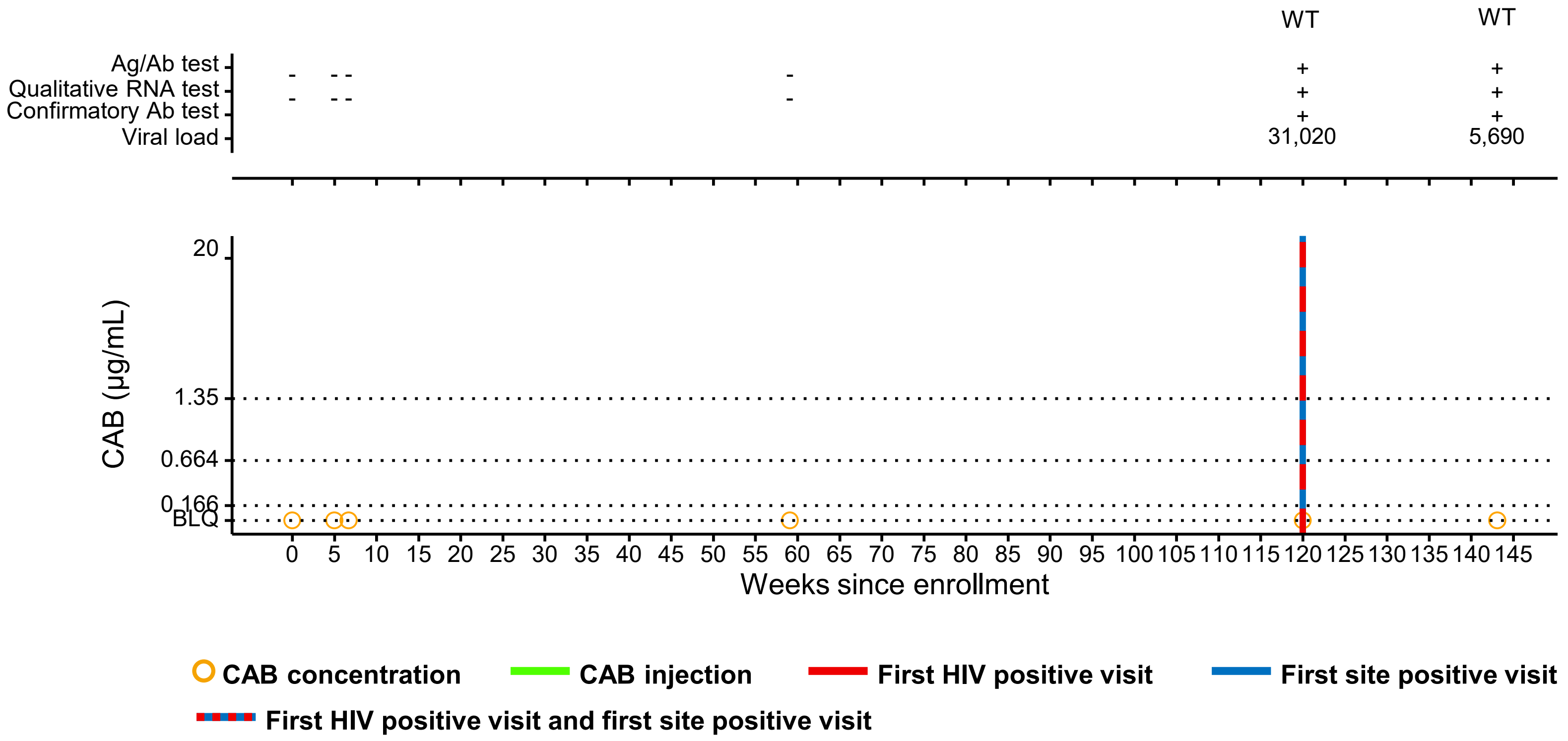

Case B8

Visit type	Diagnosis visit type	Site Testing: Rapid test 1	Site Testing: Ag/Ab test	Site Testing: DNA test	Site Testing: Viral load	Site Testing: Confirmatory Ab test	HPTN LC Testing: Ag/Ab test	HPTN LC Testing: Qualitative RNA test	HPTN LC Testing: Confirmatory Ab test	HPTN LC Testing: Viral load	HPTN LC Testing: Resistance
Week 19	1st SITE pos	NR	R		ND		NR	NR		SCA Neg	
Week 19		NR	NR	ND	ND	IND	NR	NR		SCA Neg	
Week 25		NR	NR	Detect, <LLOD	ND	IND	NR	NR		SCA Neg	
F/U Week 12								NR		SCA Neg	
F/U Week 24		NR	NR	ND	ND		NR	NR		SCA Neg	
F/U Week 48	1st HIV pos	R	NR		1,519,724		R	R	POS	631,510	L74I

HIV subtype B

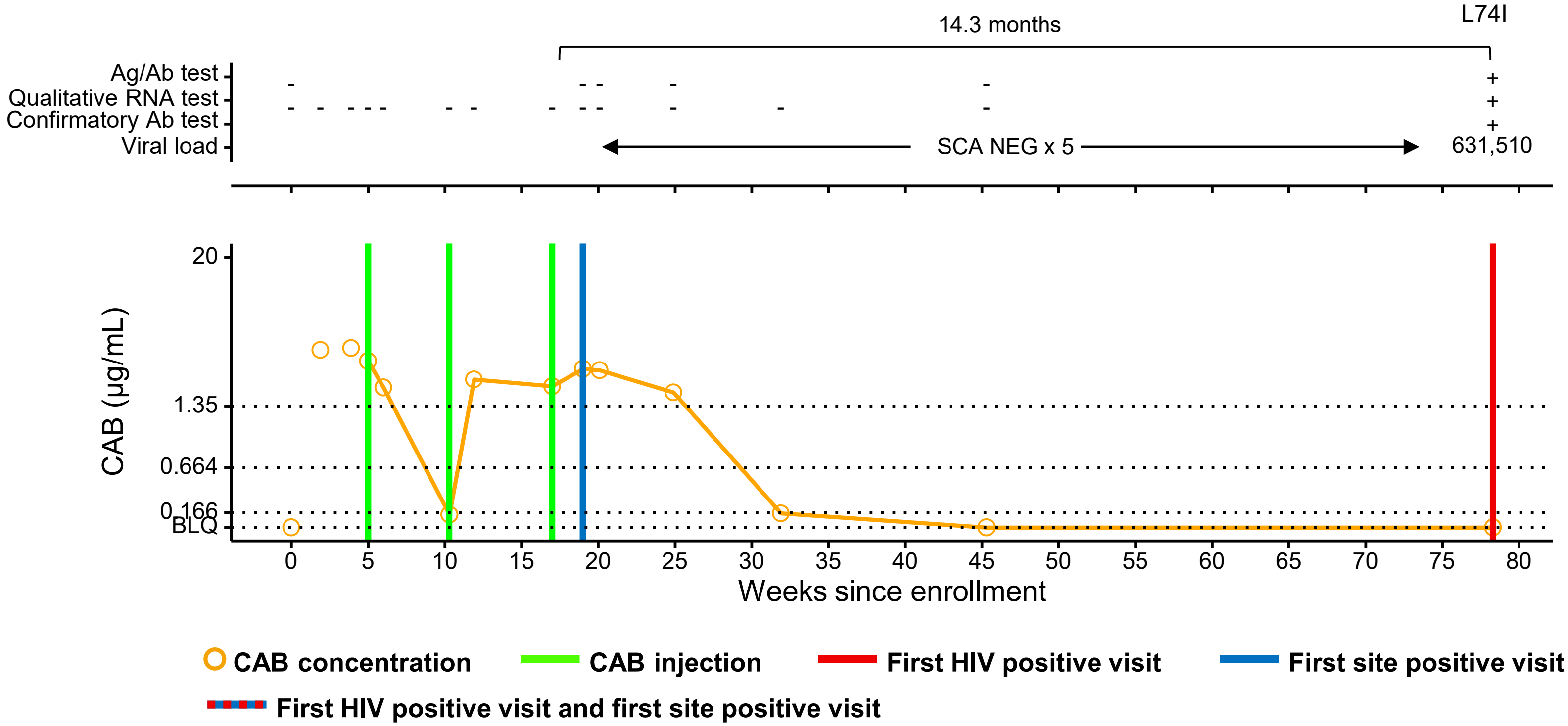

Case B9

Visit type	Diagnosis visit type	Site Testing: Rapid test 1	Rapid test 2	Ag/Ab test	Viral load	Confirmatory Ab test	HPTN LC Testing: Ag/Ab test	Qualitative RNA test	Confirmatory Ab test	Viral load	Resistance
Week 81	1st HIV/SITE pos	R	R	R	1,330,424	POS	R	R	POS	1,387,280	L74I
Interim visit		R	R	R	1,195,370	POS	R	R	POS	1,141,430	L74I
F/U Week 12**							R	R	POS	994,520	L74I
F/U Week 24					2191					2,320	
F/U Week 36										167	

**Started ART with TDF/3TC/EFV

HIV subtype B

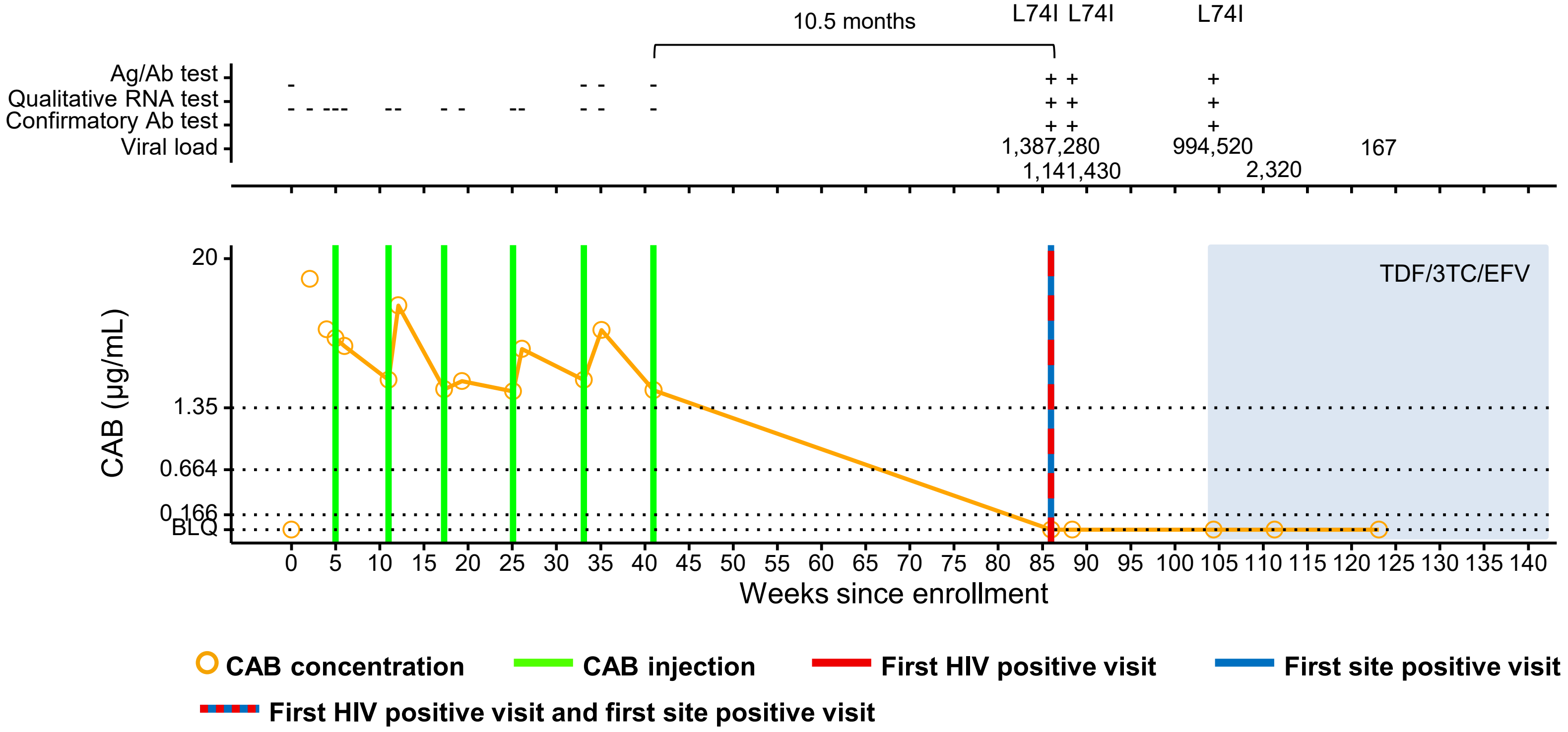

Case B10

Visit type	Diagnosis visit type	Site Testing: Rapid test 1	Site Testing: Ag/Ab test	Site Testing: Viral load	Site Testing: Confirmatory Ab test	HPTN LC Testing: Ag/Ab test	HPTN LC Testing: Qualitative RNA test	HPTN LC Testing: Confirmatory Ab test	HPTN LC Testing: Viral load	HPTN LC Testing: Resistance
F/U Week 24	1st HIV/SITE pos	NR	R			R	R	NEG	154,820	WT
Interim visit		NR	R	>10,000,000		R	R	NEG	5,013,250	WT
F/U Week 36						R	R	POS	62,440	WT
F/U Week 48**				69,257		R	R	IND	70,950	WT
Interim visit									<40	
Interim visit				<40					<40	

**Started ART with TDF/3TC/ATVr

HIV subtype AE

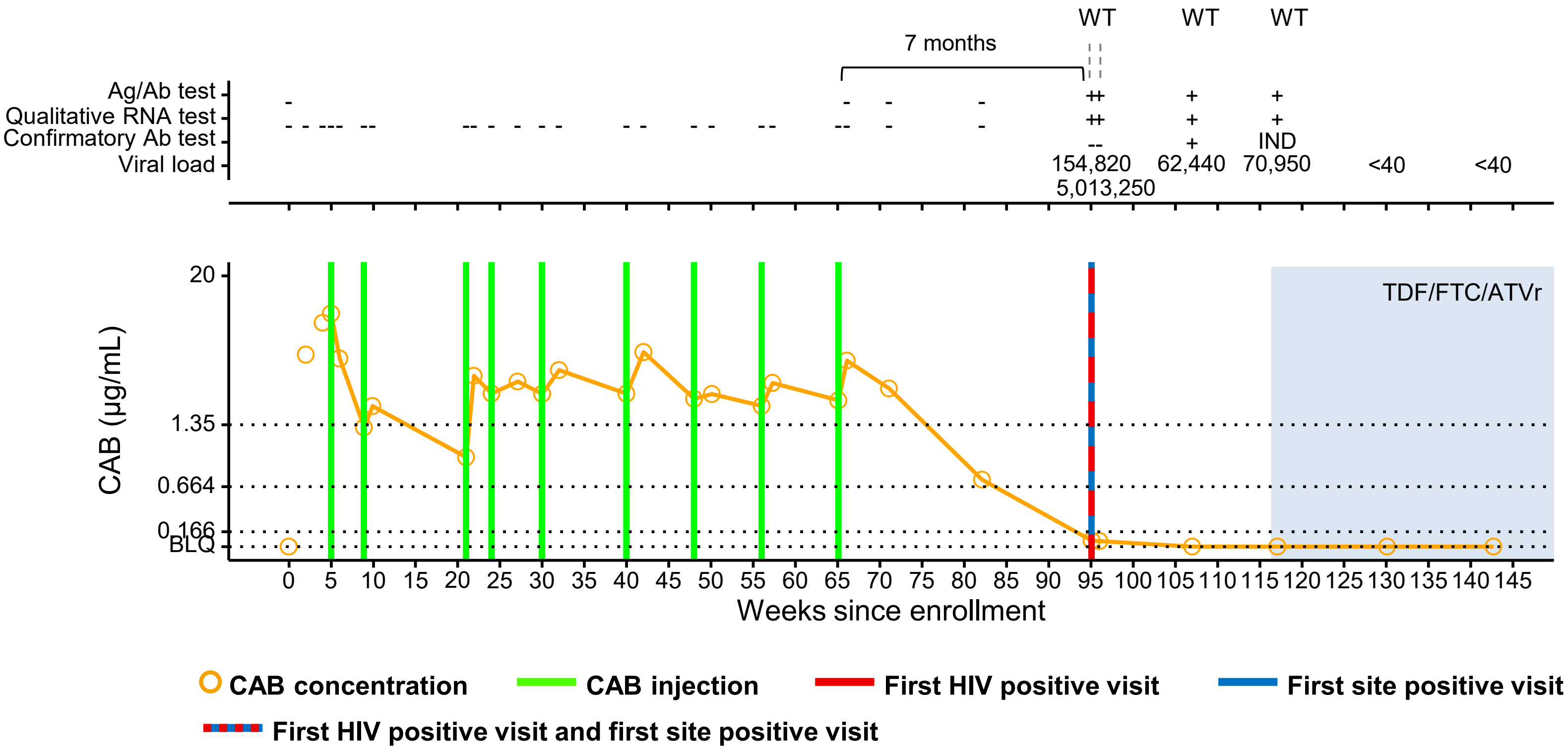

Case B11

Visit type	Diagnosis visit type	Site Testing: Rapid test 1	Rapid test 2	Ag/Ab test	Viral load	Confirmatory Ab test	HPTN LC Testing: Ag/Ab test	Qualitative RNA test	Confirmatory Ab test	Viral load	Resistance
Week 105	1st HIV/SITE pos	R	R	R	32,036	POS	R	R	POS	43,720	L74I; **K103N**
Interim visit		R	R	R	3,182	POS	R	R	POS	25,180	L74I; **K103N**
F/U Week 24**					8,920					27,100	L74I; **K103N**

**Started ART with TDF/FTC/EFV 17 days later

HIV subtype B

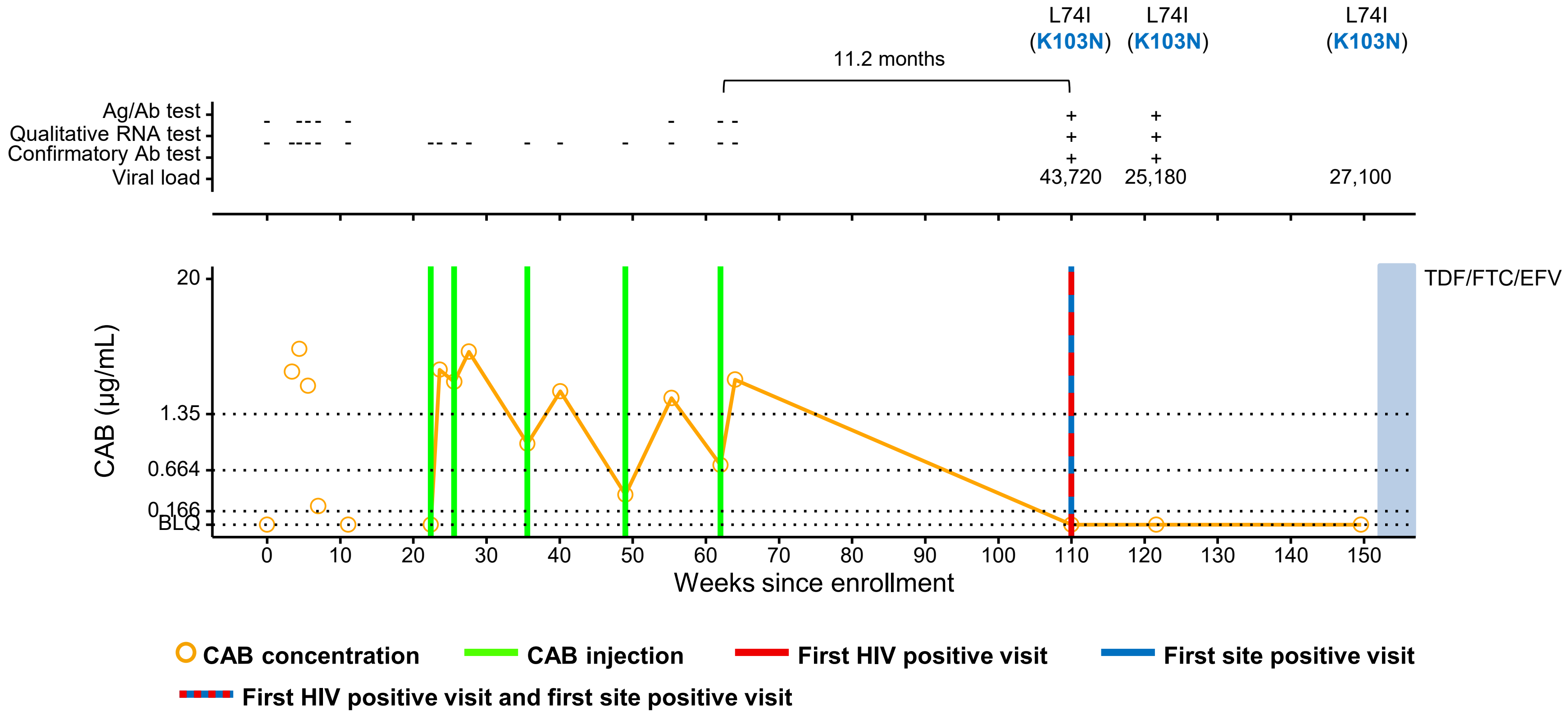

Case B12

		Site Testing					HPTN LC Testing				
Visit type	Diagnosis visit type	Rapid test 1	Rapid test 2	Ag/Ab test	Viral load	Confirmatory Ab test	Ag/Ab test	Qualitative RNA test	Confirmatory Ab test	Viral load	Resistance
Annual**	1st HIV/SITE pos	R	R	R	1,769		R	R	POS	2,330	WT

**Started ART with TDF/FTC/EFV 74 days earlier

HIV subtype B

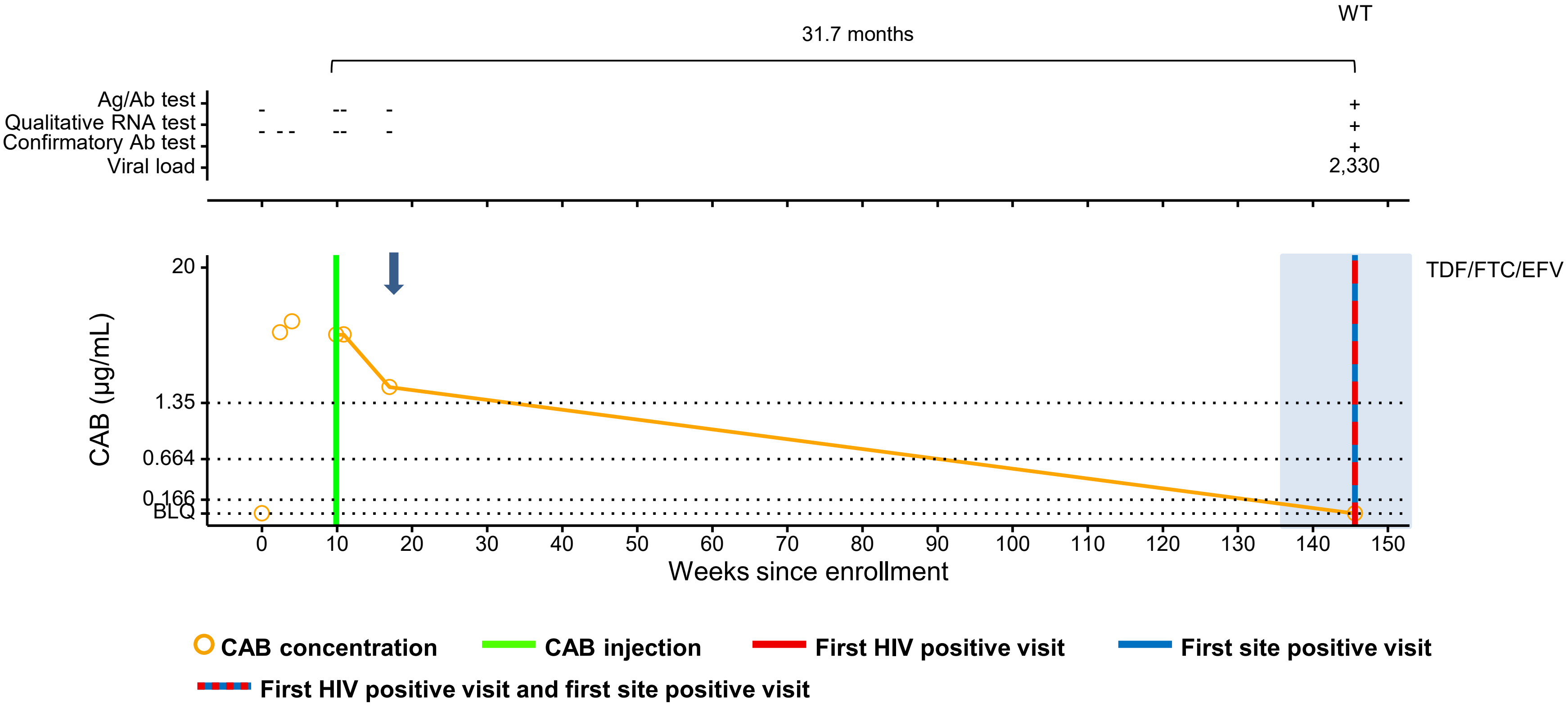

Case B13

Visit type	Diagnosis visit type	Site Testing: Rapid test 1	Site Testing: Ag/Ab test	Site Testing: Viral load	Site Testing: Confirmatory Ab test	HPTN LC Testing: Ag/Ab test	HPTN LC Testing: Qualitative RNA test	HPTN LC Testing: Confirmatory Ab test	HPTN LC Testing: Viral load	HPTN LC Testing: Resistance
F/U Week 24	1st HIV/SITE pos	R	R	54,200	POS	R	R	POS	21,290	WT
Interim visit**				<20	POS	R	R	POS	5,010	
Interim visit				ND						
OLE Week 12				165					ND	
OLE Week 36				ND					<400	

**Started ART with TAF/FTC/BIC 1 day later

HIV subtype B

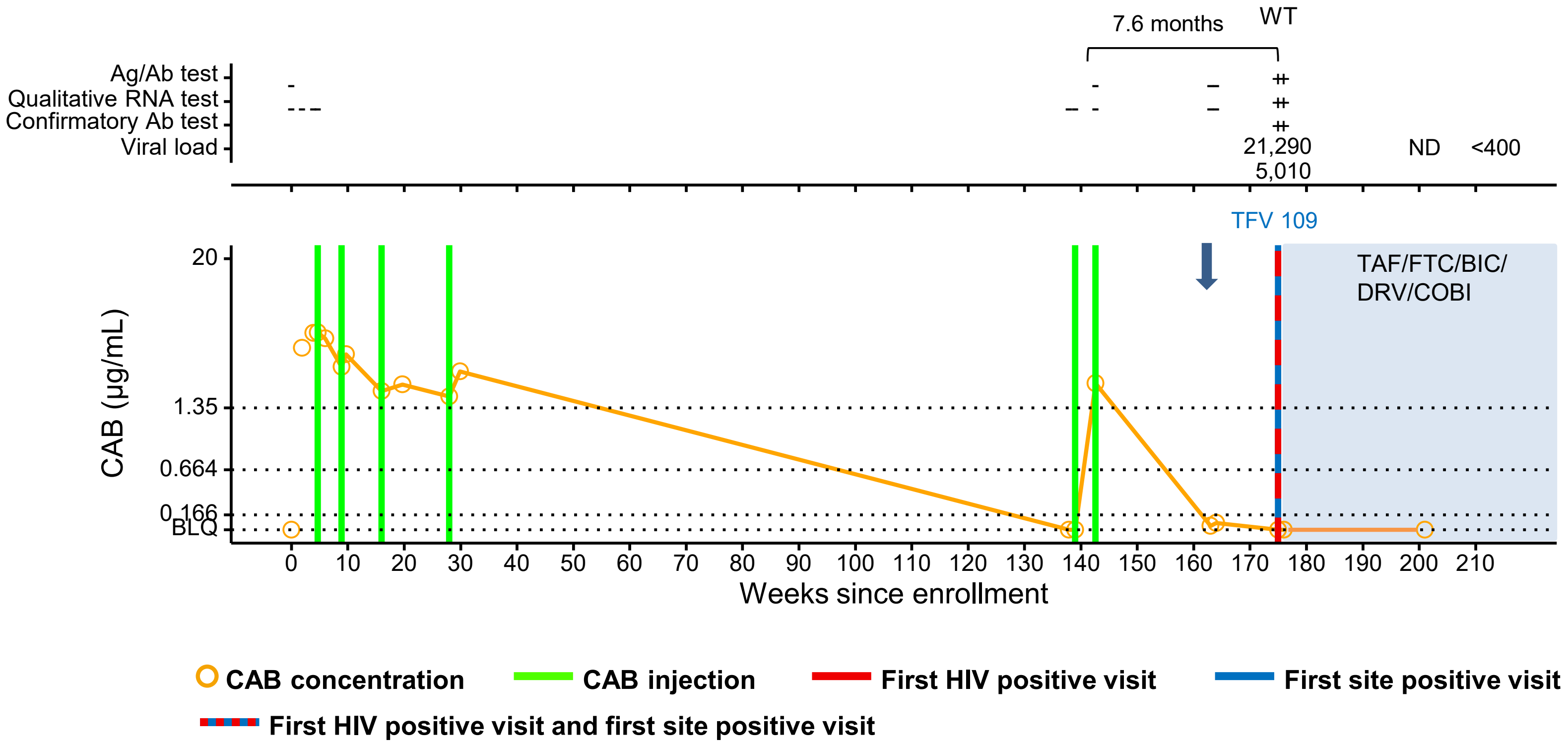

Case B14

Visit type	Diagnosis visit type	Site Testing: Rapid test 1	Rapid test 2	Ag/Ab test	Viral load	Confirmatory Ab test	HPTN LC Testing: Ag/Ab test	Qualitative RNA test	Confirmatory Ab test	Viral load	Resistance
F/U Week 24	1st HIV/SITE pos	R	R	R	134,000		R	R	POS	139,970	WT
Interim visit**		R		R	113,000	POS	R	R	POS	46,450	WT
F/U Week 36										ND	
F/U Week 48										ND	

**Started ART with TAF/FTC/BIC 50 days later

HIV subtype B

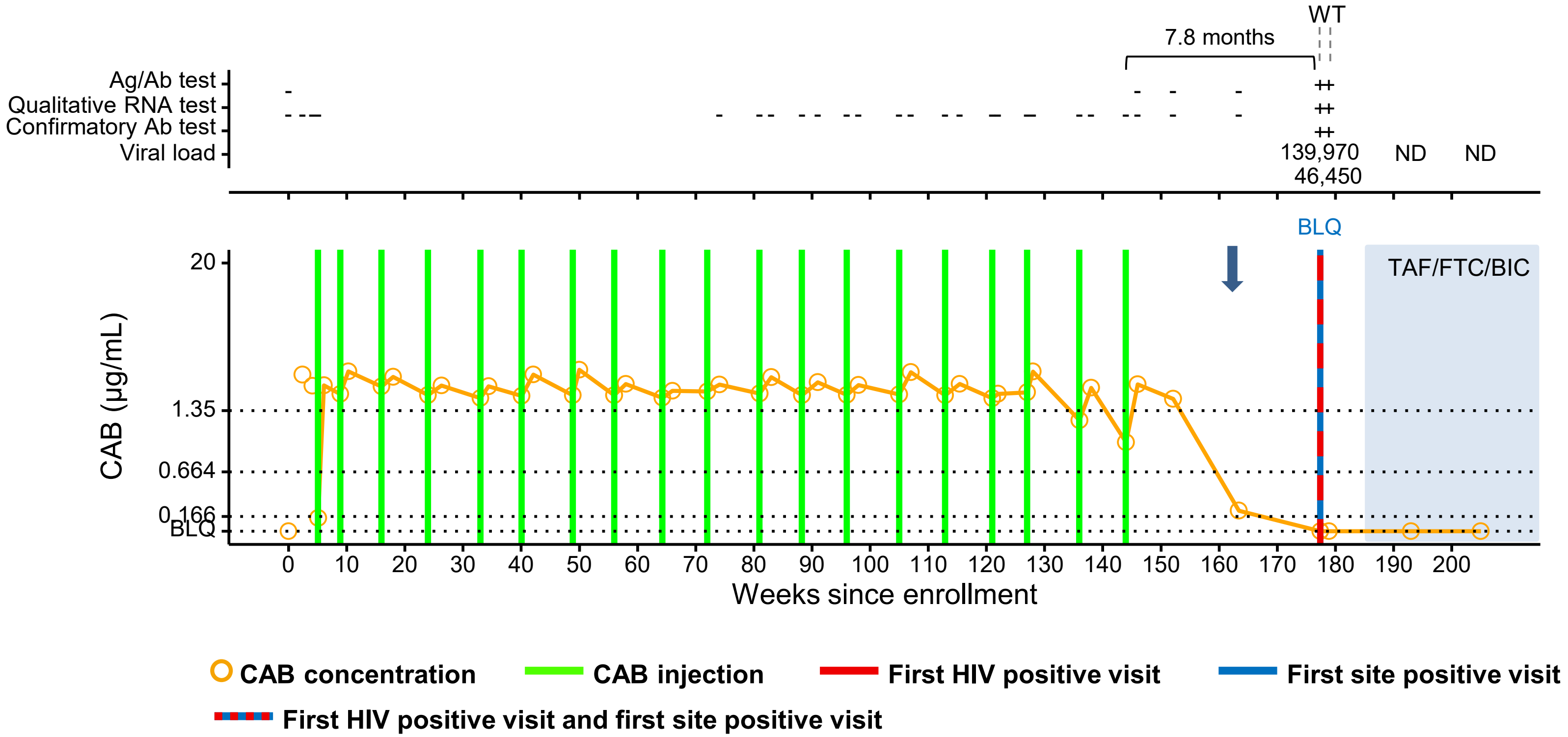

Case B15

Visit type	Diagnosis visit type	Site Testing: Rapid test 1	Site Testing: Ag/Ab test	Site Testing: Viral load	Site Testing: Confirmatory Ab test	HPTN LC Testing: Ag/Ab test	HPTN LC Testing: Qualitative RNA test	HPTN LC Testing: Confirmatory Ab test	HPTN LC Testing: Viral load	HPTN LC Testing: Resistance
F/U Week 36	1st HIV pos	NR	NR			NR	R		ND	Failed
Interim visit**	1st SITE pos	R	R	14,000		R	R	POS	7,030	M50M/I
Interim visit				18,700		R	R	POS	8,010	
OLE Week 12									<40	
OLE Week 24				ND					ND	

**Started ART with TAF/FTC/BIC 5 days later

HIV subtype B

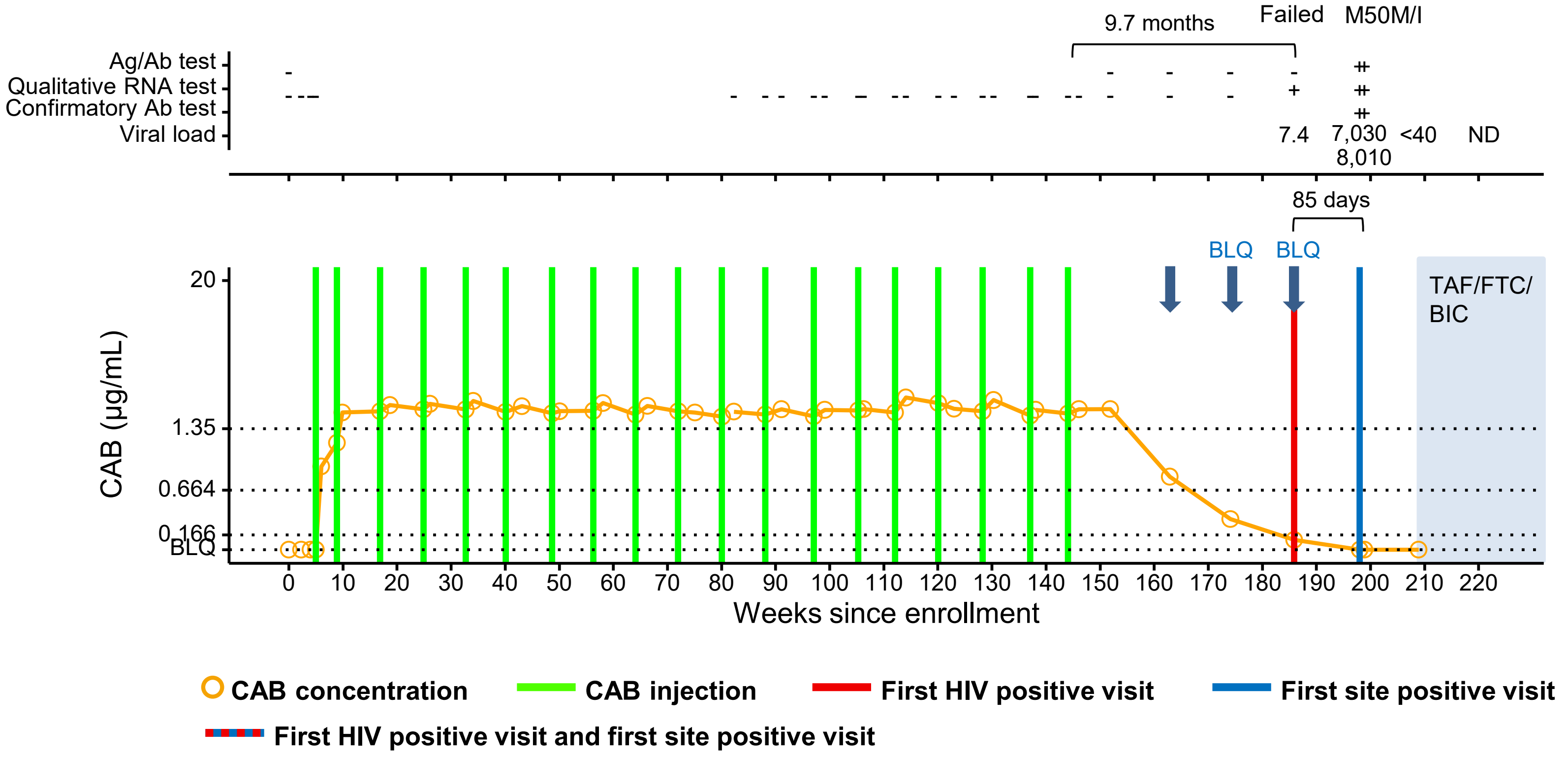

Case B16

Visit type	Diagnosis visit type	Site Testing: Rapid test 1	Site Testing: Ag/Ab test	Site Testing: Viral load	Site Testing: Confirmatory Ab test	HPTN LC Testing: Ag/Ab test	HPTN LC Testing: Qualitative RNA test	HPTN LC Testing: Confirmatory Ab test	HPTN LC Testing: Viral load	HPTN LC Testing: Resistance
F/U Week 36	1st HIV/SITE pos	NR	R		NEG	R	R	NEG	167,840	WT
Interim visit		NR	R	1,500,000						
Interim visit**		NR	R	857,000	NEG	R	R	NEG	451,150	WT
Interim visit				366						
OLE Week 12				<20					ND	
OLE Week 24				ND					ND	

**Started ART with TAF/FTC/BIC

HIV subtype B

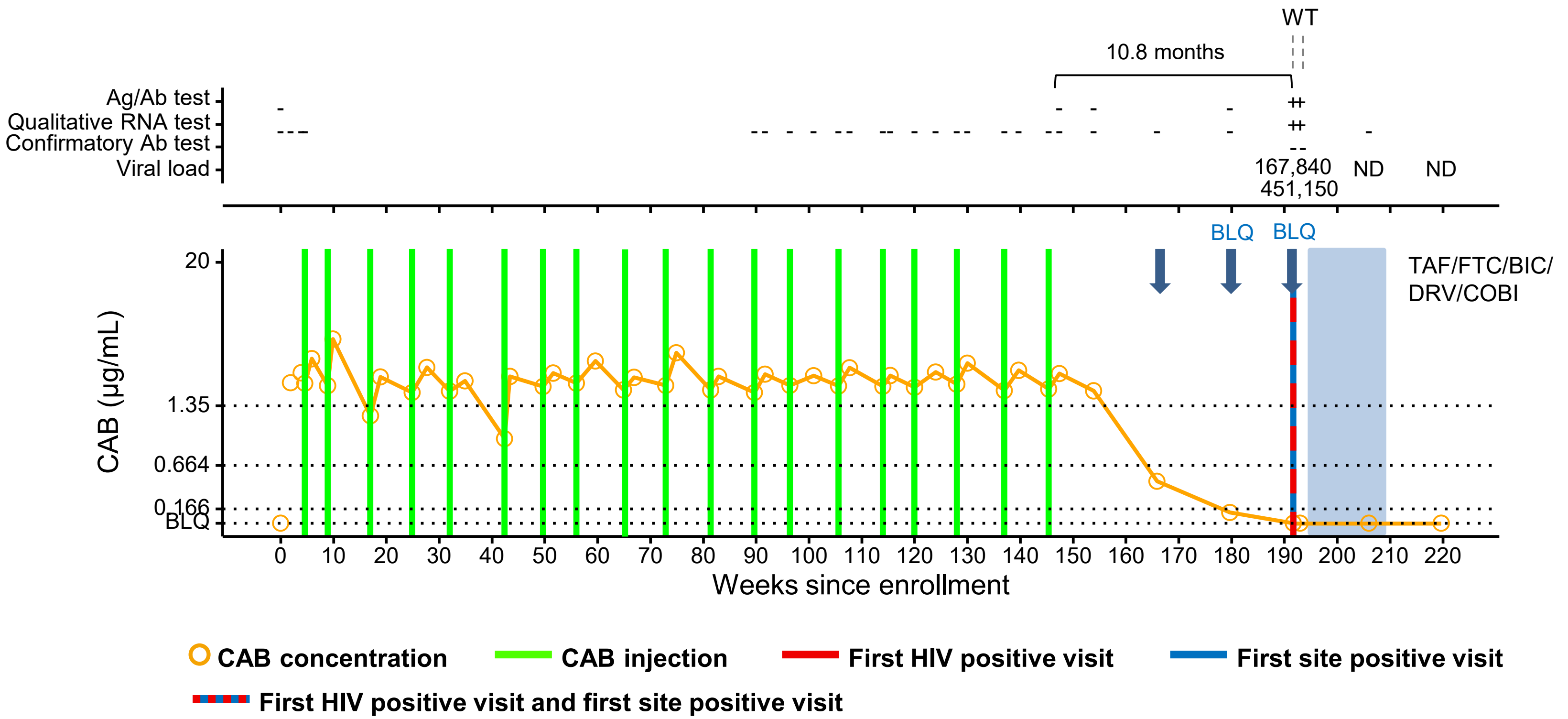

<u>Supplementary File 5. INSTI resistance summary (CAB arm)</u>

The table shows all INSTI resistance associated mutations (RAMs) detected in cases in the cabotegravir arm of HPTN 083. The mutations shown were detected at one or more study visits. Major INSTI RAMs are bolded.

ID Code	HIV Subtype	INSTI RAMs detected
A2	C	M50I, **E138K, Q148K**
A3	B	T97A
B3	AE	V151I
B6	B	M50I
B8	B	L74I
B9	B	L74I
B11	B	L74I
B15	B	M50M/I
C1	B	L74I, Q146Q/R, **E138E/K**, **G140G/S**, **Q148R**, E157Q
C3	B	**E138A**, **Q148R**
D1	Likely B	Q146L, **Q148R**, **N155H, R263K**
D2	Likely B	**N155H**, S230R
D3	BF	**R263K**
D4	C	M50I, **E138K**, **G140A**, **Q148R**
D5	F	M50I, **R263K**
D6	AE	L74I, **Q148R**
DX2	BF	V151I
BR1	BC	**Q148R**

Major INSTI RAMs were detected in 10 (31%) of the 32 CAB arm cases evaluated to date. In all ten cases, participants were received cabotegravir within six months of the first HIV positive visit (group 1, A, C, D, DX, and BR cases). Major INSTI RAMs were not detected in the 14 cases where participants had no recent CAB administration (group 2).

Accessory INSTI mutations were detected in 15 (47%) of 32 cases in the CAB arm. INSTI accessory mutations were also identified in 28 (37%) of 76 cases in the TDF/FTC arm (data not shown). The similar proportion of cases in each study arm with INSTI accessory mutations suggests that these represent baseline polymorphisms that were not acquired due to CAB exposure.

Supplementary File 6. Results from the low viral load INSTI genotyping assay.

Two samples from the 7 cases shown in Figures 1-3 were analyzed using a low viral load genotyping assay (Low VL SGS-IN). Samples selected for testing had a positive result with the APTIMA HIV-1 Qualitative Test (limit of detection: 30 copies/mL) with a viral load <500 copies/mL. The table below shows the characteristics of the samples tested and the results obtained with the Low VL SGS-IN assay. GenBank accession numbers are OP751995-OP752046.

Case	Country	Subtype	Weeks since 1st HIV pos visit	Viral load[a]	# Positive PCR reactions (%)[b]	Major INSTI RAMs[c]	INSTI accessory mutations[c]
**D5**		F	0	59	33/47 (70%)	**R263K** (11/11)	M50I (11/11)
**BR1**		BC	0	450	46/47 (98%)	None (0/38)	None (0/38)

Legend for Table.

The location of the study site (country) and HIV subtype are shown. Major mutations associated with INSTI resistance are shown in bold. Major INSTI RAMs and INSTI accessory mutations were identified using the Stanford HIV Drug Resistance Database.

[a] HIV viral load values obtained at the HPTN Laboratory Center are shown (RNA copies/mL).

[b] The table shows the number of positive PCR reactions / total PCR reactions performed per sample.

[c] The numbers in parentheses indicate number of sequences with the mutation shown per total sequences generated.

Abbreviations: 1st HIV pos visit: first HIV-positive visit; INSTI: integrase strand transfer inhibitor; ST: subtype; PCR: polymerase chain reaction; RAMs: resistance-associated mutations.
